# Structure-based assessment and druggability classification of protein–protein interaction sites

**DOI:** 10.1038/s41598-022-12105-8

**Published:** 2022-05-13

**Authors:** Lara Alzyoud, Richard A. Bryce, Mohammad Al Sorkhy, Noor Atatreh, Mohammad A. Ghattas

**Affiliations:** 1 College of Pharmacy, Al Ain University, 64141 Abu Dhabi, United Arab Emirates; 2 AAU Health and Biomedical Research Center, Al Ain University, 64141 Abu Dhabi, United Arab Emirates; 3 Division of Pharmacy and Optometry, School of Health Sciences, University of Manchester, Oxford Road, Manchester, M13 9PL UK; 4 Department of Biology, University of Toronto, Toronto, ON Canada

**Keywords:** Drug screening, Medicinal chemistry, Target identification, Chemical biology, Computational biology and bioinformatics

## Abstract

The featureless interface formed by protein–protein interactions (PPIs) is notorious for being considered a difficult and poorly druggable target. However, recent advances have shown PPIs to be druggable, with the discovery of potent inhibitors and stabilizers, some of which are currently being clinically tested and approved for medical use. In this study, we assess the druggability of 12 commonly targeted PPIs using the computational tool, SiteMap. After evaluating 320 crystal structures, we find that the PPI binding sites have a wide range of druggability scores. This can be attributed to the unique structural and physiochemical features that influence their ligand binding and concomitantly, their druggability predictions. We then use these features to propose a specific classification system suitable for assessing PPI targets based on their druggability scores and measured binding-affinity. Interestingly, this system was able to distinguish between different PPIs and correctly categorize them into four classes (i.e. very druggable, druggable, moderately druggable, and difficult). We also studied the effects of protein flexibility on the computed druggability scores and found that protein conformational changes accompanying ligand binding in ligand-bound structures result in higher protein druggability scores due to more favorable structural features. Finally, the drug-likeness of many published PPI inhibitors was studied where it was found that the vast majority of the 221 ligands considered here, including orally tested/marketed drugs, violate the currently acceptable limits of compound size and hydrophobicity parameters. This outcome, combined with the lack of correlation observed between druggability and drug-likeness, reinforces the need to redefine drug-likeness for PPI drugs. This work proposes a PPI-specific classification scheme that will assist researchers in assessing the druggability and identifying inhibitors of the PPI interface.

## Introduction

Nearly every biological function within our body is mediated by proteins. Proteins do not function in isolation; they are powered by the interactions they form with other proteins and molecules^1^. Protein interactions within cells modulate various physiological and pathological processes associated with health, constituting the human interactome network^2^. Moreover, anomalous protein–protein interactions (PPIs) and disordered proteins disrupt these intricate interactions, resulting in diseases like cancer and CNS, infectious or autoimmune disorders^3^. PPIs make up some of the most interesting yet challenging biological targets for drug discovery projects.

The core of any successful drug discovery project targeting a PPI lies within the nature of its interface and the druggability of associated binding pockets^4^. In this context, druggability refers to the likelihood of a drug-like compound to modulate or inhibit an interaction between two proteins^5^. According to Cheng et al.^6^, an estimated 60% of drug discovery projects failed due to the undruggability of the target binding site and consequent inability to bind small drug-like molecules. PPIs are often considered as undruggable or difficult targets due to their large, shallow binding interface which lacks distinct, tractable concave pockets^7^. In addition, while traditional drug targets like enzymes, G protein-coupled receptors and ion channels, fortunately have endogenous ligands that act as a starting point for these drug discovery projects, this however is not the case for PPI targets^8^. With this in mind, designing inhibitors for PPI targets can carry a substantial risk of failure. The research program Illuminating the druggable genome^9^ (IDG) has aided the deciphering of the human genome, allowing for identification of some high-potential molecular targets for drug discovery. So far only 30% of screened PPIs have been found to have potentially druggable binding sites^4,10^. This, combined with the myriad of unsuccessful attempts at developing orally available inhibitors, has prompted an argument that these potentially high-value targets are difficult^11^.

**Figure 1:**
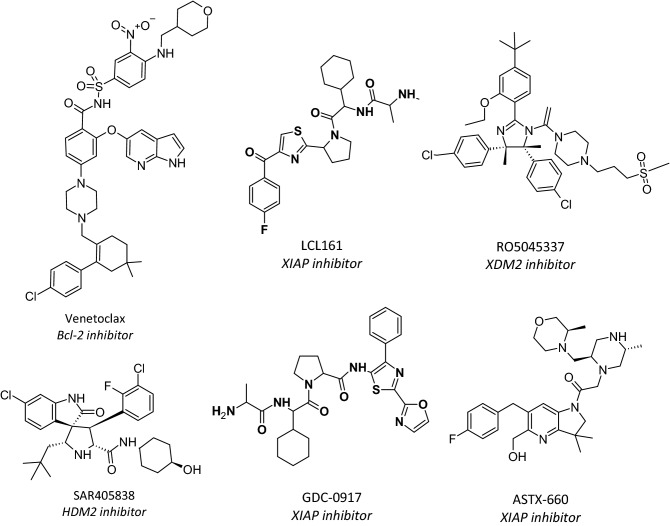
Examples of potent small molecule protein–protein interaction inhibitors that reached the clinical trials.

Over the last decade, numerous small molecule ligands have been developed to bind directly onto the PPI interface, proving that certain PPIs can accommodate small molecule inhibitors^4^ as shown in Fig. 1. Some of the aforementioned inhibitors have advanced into human clinical trials: for example, Bcl-2 inhibitor Venetoclax (ABT-199) was the first PPI drug to receive FDA approval and is now widely used in the treatment of chronic lymphocytic leukemia^12^. This suggests that once notoriously undruggable PPI interfaces have revealed a certain ability to accommodate drug-like ligands. It was shown that hydrophobic grooves on the PPI interface, where the partner proteins bind and form stable interactions, can be utilized as hot spots in the design of small molecule PPI inhibitors^13^.

Another approach for modulating PPI interactions is through PPI stabilizers^14^. These small molecules stabilize the protein–protein complex by targeting the pocket formed at the interface of two proteins^15^. In 2021, approximately 15 targeted protein degraders and molecular glues have entered the market including orally bioavailable protein degrader CFT7455^16^. CFT7455 is a novel degrader of the IKZF1/3 complex used in the treatment of Multiple myeloma and non-Hodgkin's lymphoma^17^. So far, PPI stabilizers have shown to be successful in modulating PPIs with high selectivity, allowing us to target otherwise *undruggable* protein complexes^18^. Nonetheless, the development of novel PPI stabilizers is impeded by the lack of knowledge of the mechanisms and principles of these three-body systems^18^.

In recent years, multiple in silico approaches have been developed to predict the druggability of PPI interfaces^5^. Pock-etQuery^19^, SiteMap^20^, fPocket^21^, DoGSiteScorer^22^ and SiteFinder^23^ are some of the most popular druggability assessment servers. Despite the fact that these methods have shown an ability to successfully identify druggable pockets, the majority of them do not provide a ranking or classification system for those identified pockets. SiteMap^20^ stands out as one of the most reliable algorithms to assess the druggability of biological targets, having previously been used to evaluate the druggability of protein families such as NUDIX hydrolases, Human DNA Glycosylases and bromodomains^24–26^. Here, the druggability of the site is quantified by assigning a Druggability score (Dscore), hence evaluating its potential from a drug discovery perspective.

To interpret Dscores, Halgren^27^ developed a classification system for SiteMap^20^, suggesting a cutoff point to distinguish druggable sites from difficult sites. Based on a validation set of 538 protein complexes, sites with a Dscore less than 0.8 were classified as difficult sites while protein sites with a Dscore greater than 1 were considered very druggable. However, the majority of these complexes were protein–ligand complexes, with MDM2/p53 being the only PPI included. Considering the need for a larger sample of PPIs and the numerous structural distinctions between PPI and protein–ligand interaction binding sites, it is unclear whether Halgren’s classification system^27^ can adequately describe the druggability of PPI interfaces. Modification of Dscore has been attempted previously, leading to Dscore+^11^, a score optimized based on a set of PPIs; however, this again only sets a cutoff for druggability scoring and does not offer a PPI-specific classification system. Moreover, Dscore+^11^ was not implemented within the SiteMap module. In this study, we rather focus on the direct application of Dscore to PPIs, proposing a PPI-focused classification system.

We use SiteMap to assess the protein binding interface of 320 crystal structures from 12 protein–protein interaction targets, and we introduce a new PPI-focused druggability ranking system based on Dscore. We also investigate the influence of Dscore parameters, drug-likeness, and protein flexibility on PPI druggability. The new classification system highlights the characteristic attributes of protein–protein interfaces and uses them to provide improved druggability predictions of novel PPI targets.

## Results and discussion

### Compiling a dataset of protein–protein interaction targets

The majority of druggability prediction tools, including popular servers SiteMap^20^ and Fpocket^21^, rely on Cheng et al.’s data set^6^ for validation and assessment. Nonetheless, Cheng et al.’s data set included only one PPI (HDM2, PDB code 1RV1), making it is rather unrepresentative of PPIs as a class of targets.

The Wells^28^ set has long been used to validate PPI-focused computational programs like PocketQuery^19^ and PPIMpred^29^. It does, however, have a relatively small number of targets. Recent discoveries have resulted in the identification of novel PPIs as well as orthosteric PPI inhibitors/stabilizers. Hence, new PPI databases have been curated to include additional PPI targets of high importance. The 2P2Idb^30^ database expands on the Wells set to include additional PPI targets with documented clinical implications.

As our goal was to define the parameters that guide the druggability assessment of binding sites on the PPI interface, we had to tailor the selection criteria for our data set to include high-resolution X-ray crystallographic structures of PPIs with established clinical implications. Given the unique nature of proteins, the availability of both (i) ligand-bound and (ii) protein/peptide-bound or apo-form crystal structures of a protein target is crucial for comprehensive and accurate assessment of druggability. Structures that exist as homodimers or bind covalent inhibitors were excluded from our analysis since they were beyond the scope of our research. Finally, each complex was visually inspected to ensure that the ligands bind directly to the PPI interface rather than distal locations on the protein.

The final PPI dataset included 320 hand-curated protein crystal structures belonging to 12 PPI targets: DCN1, Bcl-xL, HDM2, XDM2, Bcl-2, MDMX, VHL, HPV E2, Menin, ZipA, IL-2, and XIAP (Table 1). While the majority of these targets are associated with cancer pathogenesis, such as Bcl-xL, HDM2, XDM2, Bcl-2, MDMX, HPV E2, Menin, and XIAP; others, such as DCN1, IL-2, VHL, and ZipA, play major roles within disease pathways, resulting in the growth of non-cancerous tumors, autoimmune diseases and bacterial infections^3,31–33^.

**Table 1:** The final PPI dataset containing 320 hand-curated protein crystal structures belonging to 12 PPI targets.

PPI complex	Protein	Number of crystal structures			
		Total	Ligand-bound	Protein/peptide-bound	Apo
DCN1/UBC12	DCN1	10	NA	2	8
Bcl-xL/BAD/BAK	Bcl-xL	24	4	4	16
HDM2/p53	MDM2	87	1	22	64
XDM2/p53	XDM2	11	NA	1	10
Bcl-2/Bax/BAD	Bcl-2	26	NA	9	17
MDMX/p53	MDMX	21	NA	11	10
HPV E2/HPV E1	HPV E2	2	1	NA	1
Menin/MLL	Menin	34	1	4	29
VHL/HIF-1A	VHL	36	NA	5	31
IL-2/IL-2Rα	IL-2	14	4	4	6
XIAP/Caspase-9/Smac	XIAP	49	2	15	32
ZipA/Fitz	ZipA	6	1	1	4

*NA* Not available.

Our final dataset contains twice as many PPI targets and a significantly larger number of crystal structures than Well’s^28^ and Loving’s^11^ datasets used to study and assess PPIs. Rather than using a single representative example of ligand- and protein/peptide-bound structures to assess each target, we attempted to include as many high-resolution crystal structures as possible^34^. Expanding on commonly used PPI targets ensures that our final dataset represents a wide range of PPIs and minimizes bias when comparing in silico models.

### Druggability assessment of PPI targets using SiteMap

Druggability is a difficult concept to define because different approaches can classify sites differently. According to Cheng et al.’s definition of druggability^6^, it is the likelihood of modulating a target by drug-like molecules. To date, numerous prediction programs have been developed to aid in the identification of protein binding sites at the PPI interface; some go a step further and assess their druggability; only a few assign scores to each identified pocket. However, the majority of these tools do not provide or suggest a classification system based on their resultant scores.

To assess the druggability of PPIs, we required a tool that is readily available, reliable and allows for ligand-guided druggability estimation. More importantly, it must allow the classification and ranking of molecules included in the dataset with high accuracy^27^. This is necessary as we have identified PPI-specific druggability assessment tools that do not allow us to reach a definitive decision on which crystal structure is superior. SiteMap^20^ is one of few tools capable of identifying, assessing, scoring and classifying binding sites. In 2009, Halgren^27^ developed a robust classification system using SiteMap druggability scores (Dscore), which correctly predicted the druggability of 86% of Cheng et al.’s data set^6^. This classification system is now widely used to analyze SiteMap results. Halgren’s classification was later adopted and modified to accommodate other protein families, such as Human DNA Glycosylases^25^ and bromodomains^26^. For instance, a new class was introduced to address the marginal targets that obtain a Dscore of just less than 0.8, in order to describe those targets with marginal druggability (i.e., Moderately druggable = 0.7–0.8).

To sum up, Halgren’s classification system seems to be reasonable and robust for categorizing different targets based on their druggability; yet it needs to be refined further to address the characteristic nature of PPI interfaces, especially given that PPIs were not well-represented in the current system.

After deciding on SiteMap for druggability assessment, 320 protein crystal structures, representing a dataset of 12 PPI targets, were acquired from the Protein Data Bank (PDB). The approach adopted in this work is a ligand-guided estimation approach. Accordingly, the “evaluate single binding region” option was used to define binding pockets and direct druggability assessment towards the PPI interface. Ligand-bound structures had co-crystalized ligands, whereas apo and protein/peptide bound structures were individually superimposed to a ligand-bound structure to include a drug-like molecule as a pocket identifier. The PDB with a co-crystalized ligand that best satisfies Lipinski's drug-like rules was chosen as the reference ligand-bound structure (Supplementary Table S5). Afterward, each structure was run through SiteMap, which identified the ligand-binding site on the PPI interface and subsequently assessed its druggability. As shown in Table 2, the findings revealed that the PPI interface conveys a broad range of median Dscores (0.52–1.20). While the target DCN1 had the highest Dscore value, the protein XIAP had the lowest druggability scores among all tested PPIs; and ZipA was unable to be assessed by SiteMap since it possesses a flat interface with no well-defined pocket (Table 2).

**Table 2:** SiteMap property and Dscore values for the 12 PPIs studied. Range in parentheses. ND: binding site was not detected by SiteMap.

PPI complex	Protein	Number of crystal structures	Median Dscore	Median pocket size (*n*)	Median enclosure factor (*e*)	Median hydrophilicity factor (*p*)
DCN1/UBC12	DCN1	10	1.20 (1.14–1.25)	98 (85–110)	0.75 (0.71–0.83)	0.53 (0.46–0.66)
Bcl-xL/BAD/BAK	Bcl-xL	24	1.01 (0.38–1.17)	46 (9–194)	0.71 (0.62–0.94)	0.49 (0.12–1.17)
HDM2/p53	MDM2	87	1.00 (0.82–1.33)	54 (20–105)	0.66 (0.55–0.82)	0.28 (0.10–0.52)
XDM2/p53	XDM2	11	0.93 (0.83–1.08)	49 (27–57)	0.64 (0.62–0.73)	0.35 (0.12–0.41)
Bcl-2/Bax/BAD	Bcl-2	26	0.92 (0.74–1.19)	46 (20–104)	0.67 (0.58–0.78)	0.32 (0.15–0.53)
MDMX/p53	MDMX	21	0.86 (0.76–1.19)	40 (28–109)	0.64 (0.58–0.72)	0.33 (0.06–0.56)
HPV E2/HPV E1	HPV E2	2	0.77 (0.70–0.84)	44 (41–46)	0.68 (0.66–0.70)	0.79 (0.59–0.99)
Menin/MLL	Menin	34	0.77 (0.43–1.02)	48 (17–100)	0.72 (0.64–0.79)	0.97 (0.71–1.13)
VHL/HIF-1A	VHL	36	0.68 (0.33–0.79)	38 (8–52)	0.60 (0.56–0.64)	0.82 (0.57–1.19)
IL-2/IL-2Rα	IL-2	14	0.60 (0.35–0.80)	24 (10–55)	0.59 (0.51–0.80)	0.67 (0.43–1.15)
XIAP/Caspase-9/Smac	XIAP	49	0.52 (0.23–0.93)	27 (17–80)	0.6 (0.50–0.67)	1.01 (0.72–1.59)
ZipA/Fitz	ZipA	6	ND	ND	ND	ND

If, Halgren’s classification system^27^ was applied to this dataset. Proteins DCN1 and Bcl-xL fall into the very druggable category with median Dscores of 1.20 and 1.01 respectively. Druggable proteins such as HDM2, XDM2, Bcl-2 and MDM4 had median Dscores ranging from 0.99 to 0.86. The next four most druggable proteins, Menin, HPV E2, IL-2 and XIAP, would all be considered difficult targets by Halgren’s system as their Dscore values were shown to be less than 0.8. Based on these results, 46% of proteins in the dataset would be classified as difficult targets.

These findings raise some concerns about the appropriateness of Halgren’s^27^ classification in systems outside Cheng et al.‘s dataset, particularly when applied to PPIs. The application of this system can potentially underestimate the druggability of high-value targets protein targets. For instance, many of these PPIs proposed as difficult by the current SiteMap druggability system (Table 2) have been successfully targeted and co-crystallized with small organic molecules, and some of those have reached the clinical trials (e.g. XIAP inhibitors; ASTx-660, GDC-0917 and LCL161). Therefore, further consideration is needed to propose a new druggability classification system for PPI targets, with the aim of assisting researchers to evaluate their targets in the early stages of the drug discovery process.

### Proposed druggability classification system for the PPI interface

**Figure 2:**
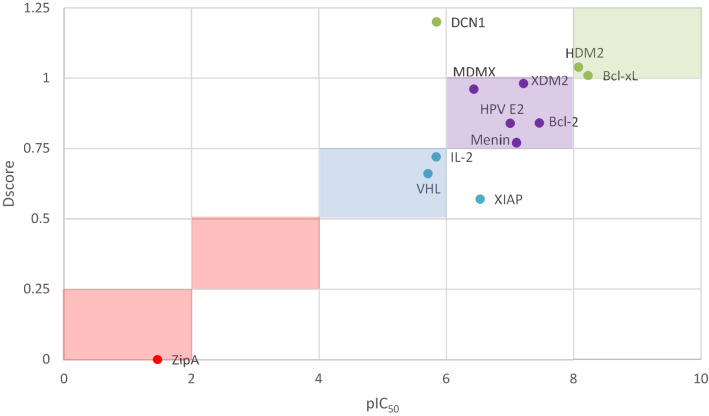
Scatter plot of mean Dscore and mean pIC50 values for each target in the proposed dataset. Dscore ≥ 1.0: very druggable (green points); Dscore ≥ 0.75 and < 1.0: druggable (purple); Dscore ≥ 0.5 and < 0.75: moderately druggable (blue); Dscore < 0.5: difficult (red). ZipA values were undefined and hence assigned a value of zero.

Consequently, we propose to adapt Halgren’s druggability scheme in a way that takes into account the characteristic nature of the PPI interface. Our proposed classification system has been validated across the 12 different protein–protein binding interfaces (Fig. 2). This system groups proteins into four main classes based on their druggability scores (Dscore): very druggable (Dscore 1.0), druggable (Dscore = 0.75 and < 1.0), moderately druggable (Dscore = 0.50 and < 0.75), and difficult (Dscore < 0.50).

Although the revised ranking is primarily based on the computed Dscore, factors such as the availability of published inhibitors and their respective binding affinity (IC_50_) were also considered in proposing the ranges for this PPI classification system. Out 139 co-crystallized protein structures, 109 ligands bind in the nanomolar levels, while another 11 ligands bind in the subnanomolar level (Supplementary Table S6). We proposed that the target should be classified based on their placement in the pIC_50_/Dscore plot (Fig. 2). This plot classifies targets similar to Halgren’s^27^, but with a slightly altered range for the druggable class and with the introduction of a new class that describes moderately druggable targets.

**Figure 3:**
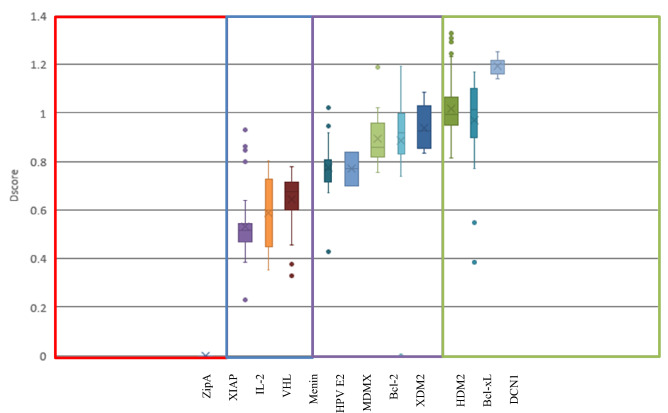
Box-plot showing range and distribution of druggability for each protein using the proposed classification system for PPIs, ranked by median Dscore. Dscore ≥ 1.0: very druggable (green box); Dscore ≥ 0.75 and < 1.0: druggable (purple); Dscore ≥ 0.5 and < 0.75: moderately druggable (blue); Dscore < 0.5: difficult (red). ZipA values were undefined and hence assigned a value of zero.

As shown in Fig. 3, the first class ‘very druggable’ represents targets that possess a mean Dscore value of greater than 1.0 (i.e. Bcl-xL, HDM2, and DCN1) and have PPI inhibitors with a mean IC_50_ value in the low nanomolar range (except DCN1, which has recently been targeted and seems to need some more time for its inhibitors to reach the nanomolar inhibition range). The second class ‘druggable’ represents targets with a Dscore range of 0.75 and less than 1.0. Interestingly, all of these PPIs showed to have inhibitors in the medium to high nanomolar inhibition range, namely Menin, MDMX, and HPVE2. The third class ‘moderately druggable’ describes targets with Dscore values ranging from 0.5 to less than 0.75 along with inhibitors in the micromolar range (i.e. IL-2, VHL, and XIAP). XIAP is an exception here as it has got a mean IC_50_ value in the nanomolar range, however, it has the least druggable pocket at all which makes it classified as moderately druggable and as challenging as the other two PPIs in this category. Finally, the ‘difficult’ category includes targets with a Dscore of less than 0.5 along with inhibitors in the millimolar (mM) range (i.e., ZipA).

**Figure 4:**
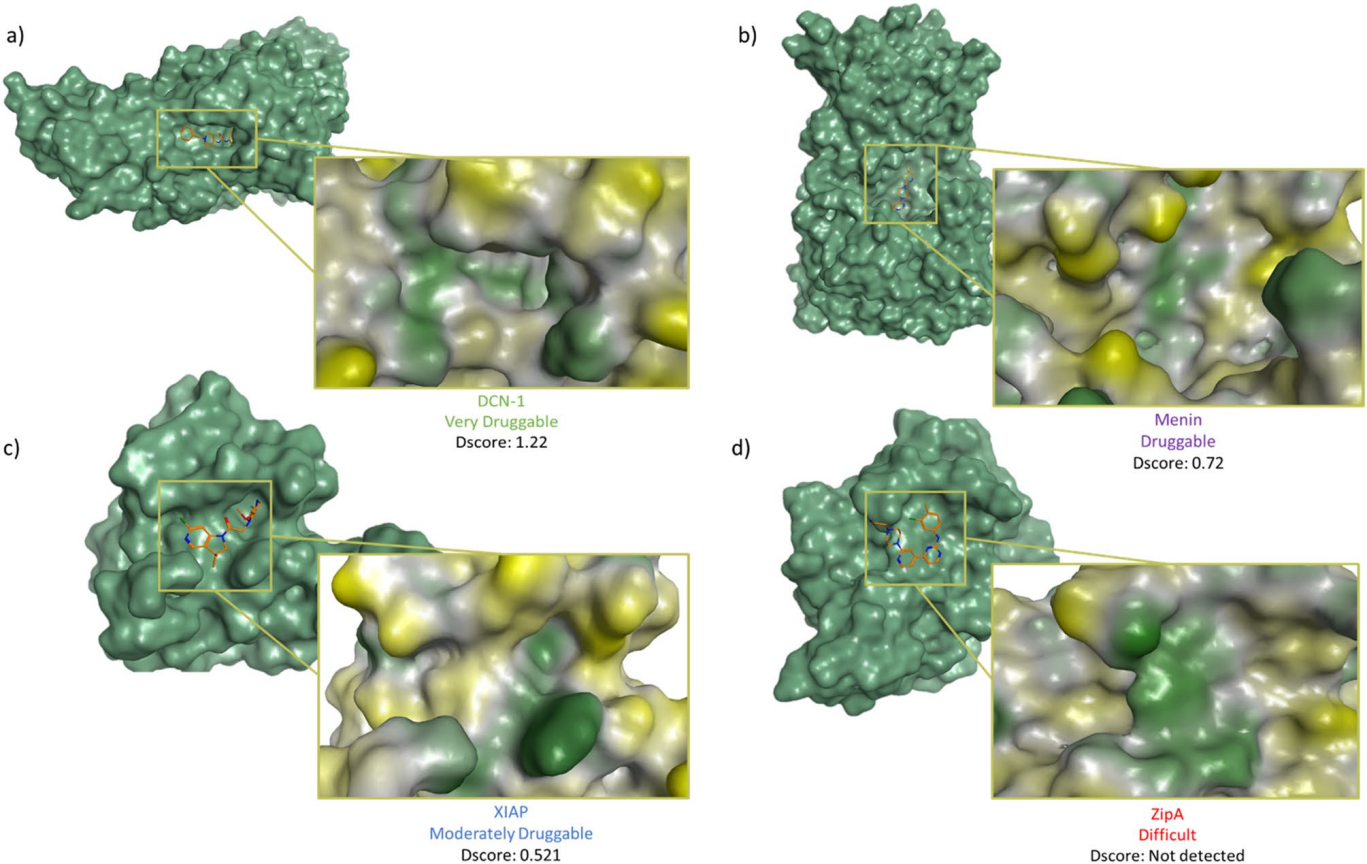
The binding sites of the four classes of PPI illustrated on the surface of a representative example. Surface colored generated using MOE Pocket coloring: green = hydrophobic, yellow = hydrophilic, and grey = neutral. (**a**) The crystal structure of DCN-1 with NAcM-HIT (PDB: 5V83^35^). (**b**) The crystal structure of Menin with MI-273 (PDB: 5DDF^36^). (**c**) The crystal structure of XIAP with compound 20 (PDB: 5C84^37^). (**d**) The crystal structure of ZipA with inhibitor DB03916 (PDB: 1Y2F^38^).

Figure 4 and Table 2 depict several distinctions in the binding interfaces between classes. The highest-ranking protein, DCN1, possesses a large pocket (n = 98 spheres) which is well-defined (e = 0.71) and with moderate hydrophilicity (p = 0.53). Similarly, another “very druggable” proteins, Bcl-xL and HDM2, have a smaller (n = 46 and 54 spheres), less defined cavity (e = 0.644 and 0.72) and lower hydrophilicity (p = 0.49 and 0.28). Note that regardless of the variation between proteins in this class, they all have inhibitors in the subnanomolar and low nanomolar ranges. The second class includes “druggable” proteins like Menin, which has a slightly smaller (n = 47.5), and more well defined pocked (e = 0.72) than HDM2, but its remarkably high hydrophilicity (p = 0.97) shifted it to a lower class than HDM2. Protein MDMX is of a smaller size (n = 40), but maintains enclosure (e = 0.64) and hydrophilicity (p = 0.33) factors within range, making it druggable. Targets with higher hydrophilicity, such as HPV E2 and Menin, have lower Dscores than more hydrophobic proteins in the same class. This is seen in the druggable proteins Bcl-2 and HPV E2, which have different hydrophilic properties (p = 0.32 and 0.79, respectively), yet both belong to the same class of proteins. The next class includes marginal targets classified as “moderately druggable” such as VHL, IL-2, and XIAP. These targets feature remarkably small pockets (n = 23–38 spheres) that are moderately enclosed (e = 0.59–0.6) and highly hydrophilic (0.67–1.01). Despite having a very small pocket (n = 27 spheres), and a shallow cavity (e = 0.6) that seems to be hydrophilic in nature (p = 1.01), several compounds have been reported to inhibit XIAP. Inhibitors ASTX660 and LCL-161 have successfully completed phase I of clinical trials, demonstrating that although this PPI target has been initially seen as challenging, it is a promising target^3^. In fact, nearly all reported inhibitors for moderately druggable targets are in the high nanomolar to micromolar ranges. No well-defined pocket was detected by SiteMap for protein ZipA, which is known for having a distinctive flat interface. As a result, ZipA was classified as a “difficult” target. This protein has a few inhibitors known in the literature; however, these do not bind to a cavity in the PPI interface, but rather to high energy hotspots on the protein^11^, which explains why efforts over the last 20 years have failed to generate a ZipA inhibitor that can reach clinical trials^13^.

Having proposed this classification system, it is still important to exercise caution when designing small molecule inhibitors for PPI targets. We believe that the druggability of a PPI target cannot be confined to an assessment of the interface alone. When thoroughly evaluating a protein binding site, several factors must be considered, including the effect of ligand binding; the flexibility of the protein; and the drug discovery history of the PPI of interest.

### Effect of Dscore parameters: size, enclosure and hydrophilicity on druggability

**Figure 5:**
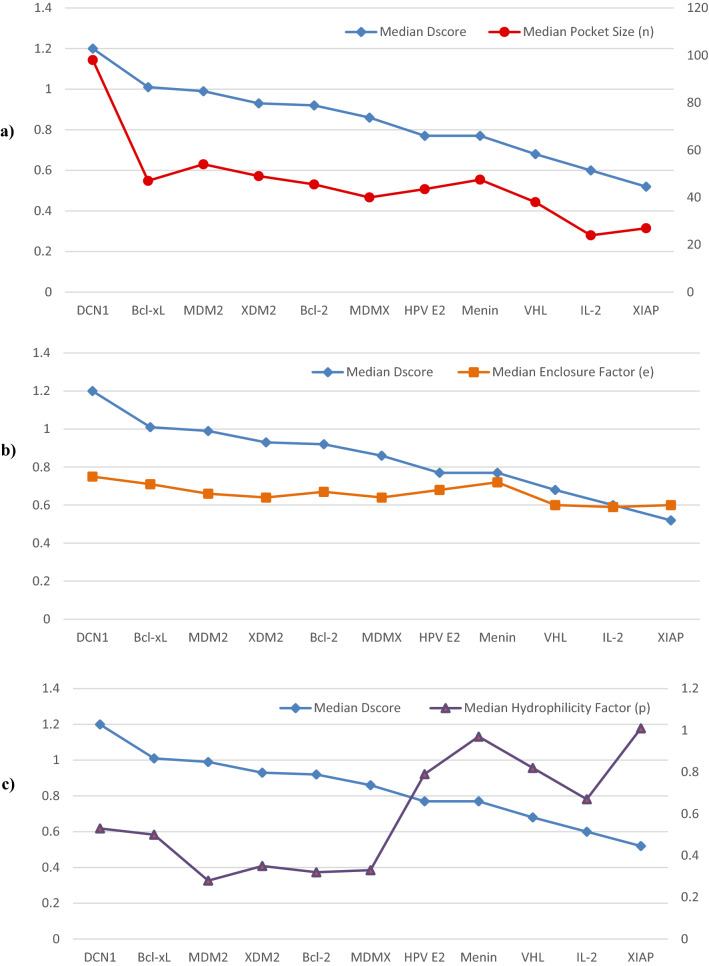
The correlation between the druggability of studied PPI and their (**a**) pocket size (**b**) enclosure and (**c**) hydrophilicity. ZipA values were undefined and hence assigned a value of zero.

SiteMap computes descriptors other than Dscore to provide further insight into the physiochemical properties of a binding site. Given that PPI targets have distinct structural features, it would be interesting to see which descriptors have the strongest correlation with the Dscore values of the PPI studied (Supplementary Fig. S1). When computing druggability via Dscore, the descriptors employed are pocket size, enclosure, and hydrophilicity. A moderate positive correlation between both pocket size, enclosure and Dscore value is observed (Fig. 5) (R^2^ = 0.74 and 0.58, respectively). In different protein binding sites, a directly proportional relationship between pocket size and Dscore is observed in Fig. 5a, with larger pockets reflecting higher druggability scores and smaller pockets reflecting lower druggability scores. Additionally, pocket enclosure is directly related to Dscore values as shown in Fig. 5b; but it has a subtle influence on druggability scores when compared to the pocket size parameter.

The degree of hydrophilicity is another parameter used in the Dscore druggability equation. It differentiates between druggable and difficult pockets by penalizing highly polar sites that are less likely to bind drug-like ligands. Figure 5c depicts an inverse relationship between pocket hydrophilicity and Dscore values (R^2^ = 0.46). This subtle effect becomes more evident as the protein shifts from one class to another; thus, as the hydrophilicity of the identified pocket increases, the Dscore decreases.

Overall, the observed correlation suggests that larger, well-defined hydrophobic pockets are more likely to bind drug-like ligands, which is reasonable (Supplementary Fig. S1). The shallowness of the pockets on the PPI interface greatly compromises its size and enclosure, and to a slightly lesser extent the hydrophilicity of the pocket.

### Druggability of apo, ligand-bound and protein/peptide bound forms of PPIs

Having assessed the druggability of the PPI dataset, we next investigate the influence of protein flexibility on the active site. Since the nature of the available crystal structures can influence binding site assessment, we separated structures into apo, ligand-bound and protein/peptide bound groups and then compared them to the overall median, median Dscore and pocket size values reported for each protein (Table 2); this provides a better understanding of the extent of conformational effects within the dataset^39^. It is worth noting that only small molecules are represented in the ligand-bound group. Whereas peptide inhibitors molecules, which bind at the PPI interface as a secondary structure, have been included in the protein/peptide bound groups. Table 3 breaks down the median druggability score (Dscore) and pocket size values for the PPI studied in this way, based on the nature of the crystal structure.

**Table 3:** Median druggability score (Dscore) and pocket size values for the 12 PPIs studied. ND indicates no data.

	Number of crystal structures			Median Dscore (± SD)			Median pocket size (± SD)		
	Apo	Protein/peptide-bound	Ligand-bound	Apo	Protein/peptide-bound	Ligand-bound	Apo	Protein/peptide-bound	Ligand-bound
DCN1	NA	2	8	NA	1.15 (0.01)	1.21 (0.03)	NA	96 (4.24)	98 (7.87)
Bcl-xL	4	4	16	0.73 (0.29)	0.87 (0.10)	1.09 (0.09)	19 (8.73)	32.5 (5.94)	140 (63.82)
HDM2	1	22	64	0.93	0.97 (0.06)	1.03 (0.11)	42	52 (11.11)	55 (16.35)
XDM2	NA	1	10	NA	0.85	0.93 (0.08)	NA	27	50.5 (7.84)
Bcl-2	NA	9	17	NA	0.84 (0.06)	0.97 (0.10)	NA	30 (11.86)	50 (17.00)
MDM4	NA	11	10	NA	0.90 (0.07)	0.85 (0.13)	NA	40 (7.27)	36.5 (24.70)
HPV E2	1	NA	1	0.70	NA	0.84	41	NA	46
Menin	1	4	29	0.79	0.88 (0.10)	0.76 (0.10)	45	71 (19.16)	46 (17.20)
VHL	NA	5	31	NA	0.62 (0.13)	0.69 (0.10)	NA	29 (13.20)	39 (10.62)
IL-2	4	4	6	0.48 (0.19)	0.48 (0.07)	0.72 (0.12)	21.5 (6.85)	19.5 (6.70)	30.5 (13.02)
XIAP	2	15	32	0.31 (0.11)	0.52 (0.04)	0.52 (0.14)	17 (0)	26 (4.26)	28 (16.35)
ZipA	1	1	4	ND	ND	ND	ND	ND	ND

Protein–ligand complexes dominated the dataset, with a total of 230 crystal structures across the 12 PPIs. We aimed to incorporate as many ligand-bound structures as possible in an effort to minimize potential errors caused by varied estimations of different pockets. With a few exceptions, analyses of peptide/protein-bound complexes yielded similar results to those of ligand-bound complexes. Proteins XIAP and XDM2 showed almost no deviation from the ligand-bound median Dscore and pocket size values (Table 3), while proteins DCN1, HDM2, Bcl-2, MDMX, and VHL deviated by less than 10%. In contrast, Menin, Bcl-xL and IL-2 were the only exceptions here, demonstrating substantial deviations from their respective ligand-bound medians. The druggability of protein Menin was increased as a result of its large pocket size in the protein-bound form (n = 71 spheres), which was significantly larger than the ligand-bound median value (n = 46 spheres). Conversely, the pocket size of both Bcl-xL and IL-2 was substantially reduced upon protein/peptide binding (n = 33 and 19.5 spheres, respectively) compared to the ligand-bound peers (n = 138 and 31 spheres, respectively), preventing them from having adequate Dscores compared to the ligand-bound median Dscores (20% and 33% reductions). For the most part, these results imply that protein/peptide bound complexes tend to yield comparable induced-fit conformational changes in the PPI interface to what we see in the ligand-bound complexes.

Apo structures were the least abundant, accounting for less than 5% of the PPI dataset. The apo structures exhibited the greatest percentage of deviations from the ligand-bound Dscore median. Interestingly, Bcl-xL and XIAP showed a pronounced reduction in druggability, with decreases in median Dscore of 33% and 40% respectively. This is mainly attributed to their very small pocket size (of 17 and 19 spheres, respectively), which are unlikely to accommodate drug-like small molecules. IL-2 exhibited a pocket structure similar to its peptide/protein bound structure, but yet again a 33% smaller Dscore than the ligand-bound form because of the large variation in the pocket size. Lastly, apo HDM2, Menin and HPV E2 structures deviated from the overall median by less than 10%, indicating that they have comparable Dscores and pocket size values. To sum up, crystal structures should be analyzed carefully especially if they have no ligand bound to the PPI interface, as they tend to show poorly druggable pockets and hence can give false conclusions about the druggability of the tested target.

### The influence of protein interface flexibility on the PPI druggability

**Figure 6:**
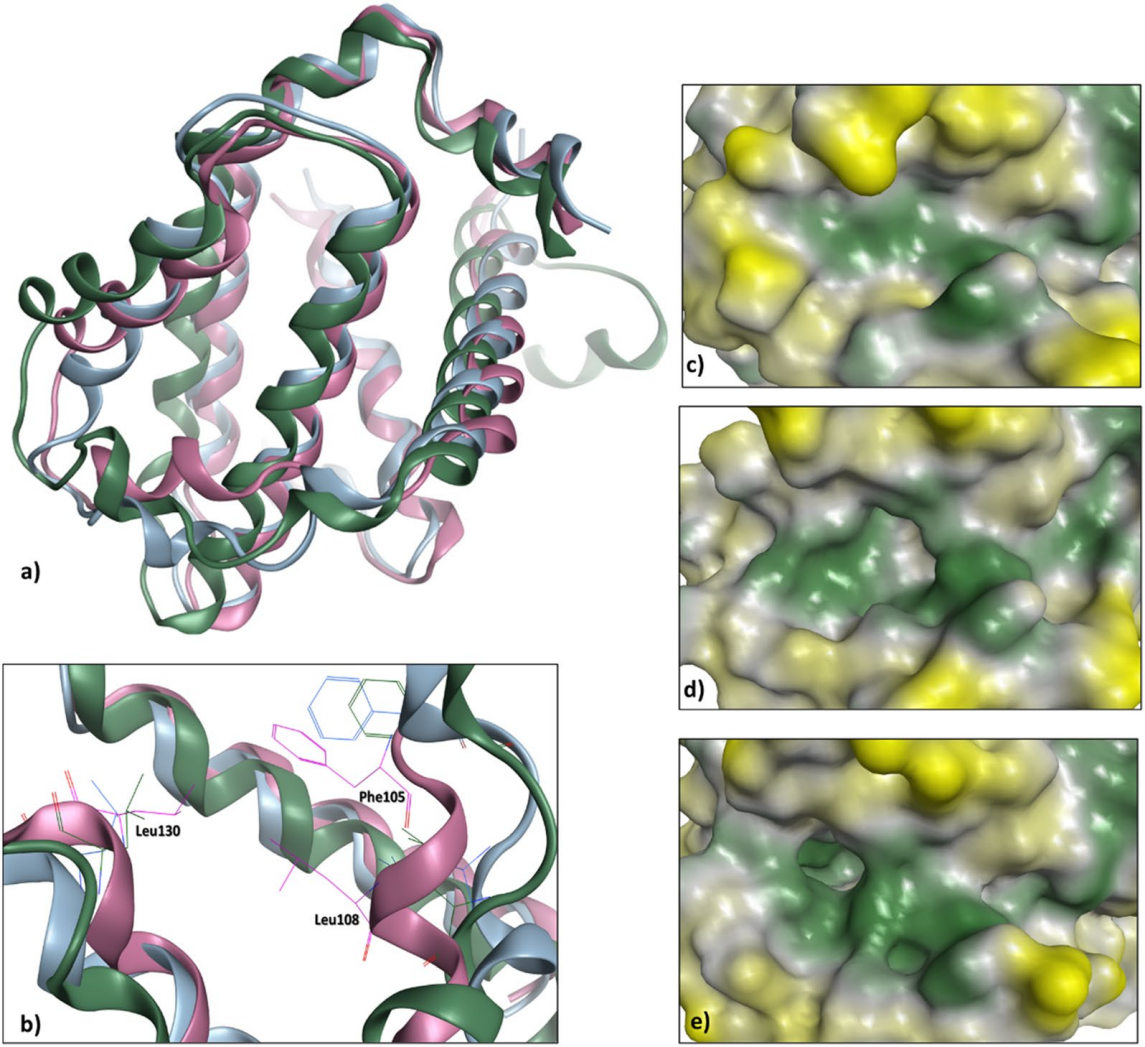
The protein binding interface of three Bcl-xL crystal structures aligned on each other. (**a**) The apo structure is shown as pink ribbons, the peptide-bound structure as blue ribbons and the ligand-bound structure as green ribbons. (**b**) Residues Phe105, Leu108 and Leu130, shown in sticks, represent conformational changes in three Bcl-xL structures. The Bcl-xL interface is clearly showing different topology when comparing (**c**) the apo form (PDB: 1R2E^40^) with (**d**) the peptide-bound form (PDB: 2BZW^41^) and (**e**) the ligand-bound form (PDB: 4C52^42^), where the latter demonstrates the most well-defined pocket, particularly when compared to the apo structure. Notes: Surface color generated using MOE Pocket coloring: green = hydrophobic, yellow = hydrophilic, and grey = neutral.

**Figure 7:**
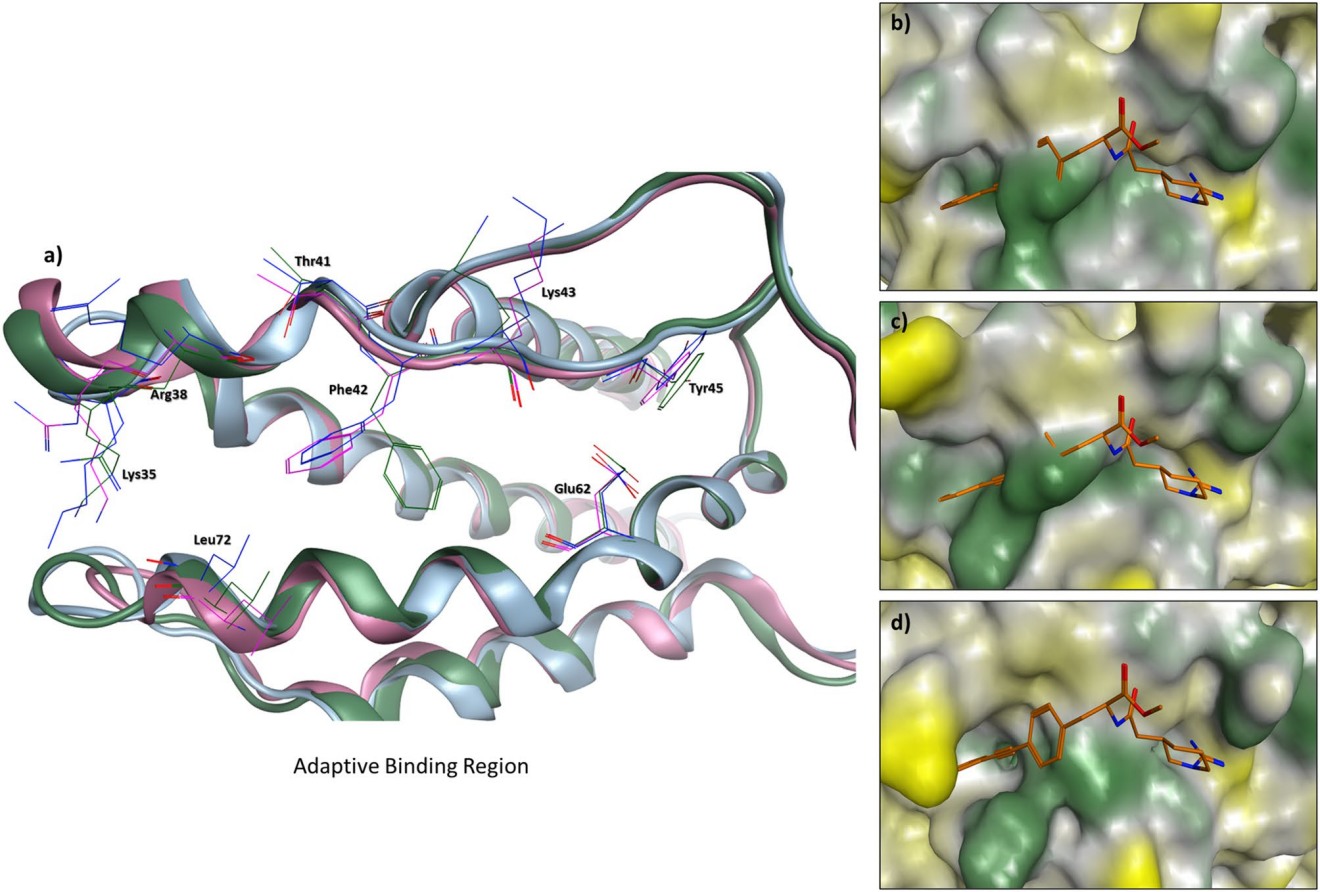
The protein binding interface of three IL-2 crystal structures aligned on each other. (**a**) Structural representation of the adaptive binding region at protein Interleukin 2 (IL-2) binding site. Apo structure is shown as pink ribbons, the peptide-bound structure as blue ribbons and the ligand-bound structure as green ribbons. It is evident that (**b**) the IL-2 apo form (PDB: 1M47^43^) and (**c**) the protein-bound form (PDB: 2ERJ^44^) completely lack the targeted binding cavity when compared to (**d**) the ligand-bound form (PDB: 1M48^43^). The co-crystallized IL-2 inhibitor, (R)-*N*-[2-[1-(Aminoiminomethyl)-3-piperidinyl]-1-oxoethyl]-4-(phenylethynyl)-l-phenylalanine methyl ester (orange sticks), was aligned on the apo and protein bound structures to emphasize the change happening in the topology of the IL-2 interface. Surface color generated using MOE Pocket coloring: Green = hydrophobic, Yellow = hydrophilic, and Grey = neutral.

PPIs are notorious for their high flexibility and chain movement^40^, which might explain the aforementioned data regarding the druggability variation found for different types within a PPI structure. Notably, several studies have analyzed the chain flexibility in the Bcl-xL family of proteins and its effect on predicted druggability^2,41,42^. When the druggability score of each conformational sample is compared, significant differences can be observed between the apo (PDB: 1R2E^43^, Dscore = 0.55), peptide-bound (PDB: 2BZW^44^, Dscore 0.85) and ligand-bound structures (PDB: 4C52^45^, Dscore = 1.08). Therefore, the analysis of the Bcl-xL apo form does not predict grooves and cavities in the protein interface which are able to bind small molecules, whereas the assessment of peptide- and ligand-bound Bcl-xL structures are successful in predicting cavities of varying features (Fig. 6). To explain this, Loving et al.^11^ proposed that Phe105 and Leu130 adopt different conformations, causing the helix surrounding Leu108 to become disordered and form a ligand binding pocket (Fig. 6). This implies that ligand binding induces conformational changes, resulting in the formation of a druggable pocket that would not occur otherwise. Another example is IL-2 which exhibited a varied druggability scoring depending on its structural state (Table 3). While there is a distinction between apo and bound structures, ligand- and peptide-bound structures might be expected to undergo similar structural changes. However, because the IL-2 protein binding interface has a highly adaptive region (Fig. 7), it is susceptible to unpredictable structural changes and thus exists in a number of different conformations^46,47^. This explains why ligand binding can produce a pocket that is 30% larger than peptide/PPI binding (Fig. 7). This adaptive interface provides an advantage because it exposes binding sites for small molecules and opens up new opportunities in drug discovery.

Examining the variation in druggability scores between different crystal structures of HDM2 and MDMX finds that all apo, protein/peptide- and ligand-bound proteins yield similar scores, with less than 10% variation. This is noteworthy because the protein interface of HDM2 and MDMX consists of a flexible N-terminal region that interacts with several proteins, including p53^48,49^. Hence, one would expect a greater variation in druggability scores between different conformations, particularly in the apo structure. However, in this case, the flexible N-terminal does not significantly open pockets on the PPI interface. This shows that, for a small number of targets, protein flexibility has a minimal effect on the final druggability score.

Moreover, the topology of the PPI interface seems to not only influenced by whether it binds a ligand or not, but also the type, size and nature of that ligand. Table 3 shows that the Dscores of the ligand-bound form of IL-2 that ranges from 0.44 to 0.80, and for the XIAP target that ranges from 0.39 to 0.93. In the latter case, the obtained scores widely varies to the extent that it spans the four druggability classes (from “difficult” to “very druggable”). Hence, selecting an appropriate crystal structure is a critical factor in obtaining accurate and representative druggability predictions, especially when dealing with a PPI target that is known for its dynamic structure and highly adaptive interface.

**Figure 8:**
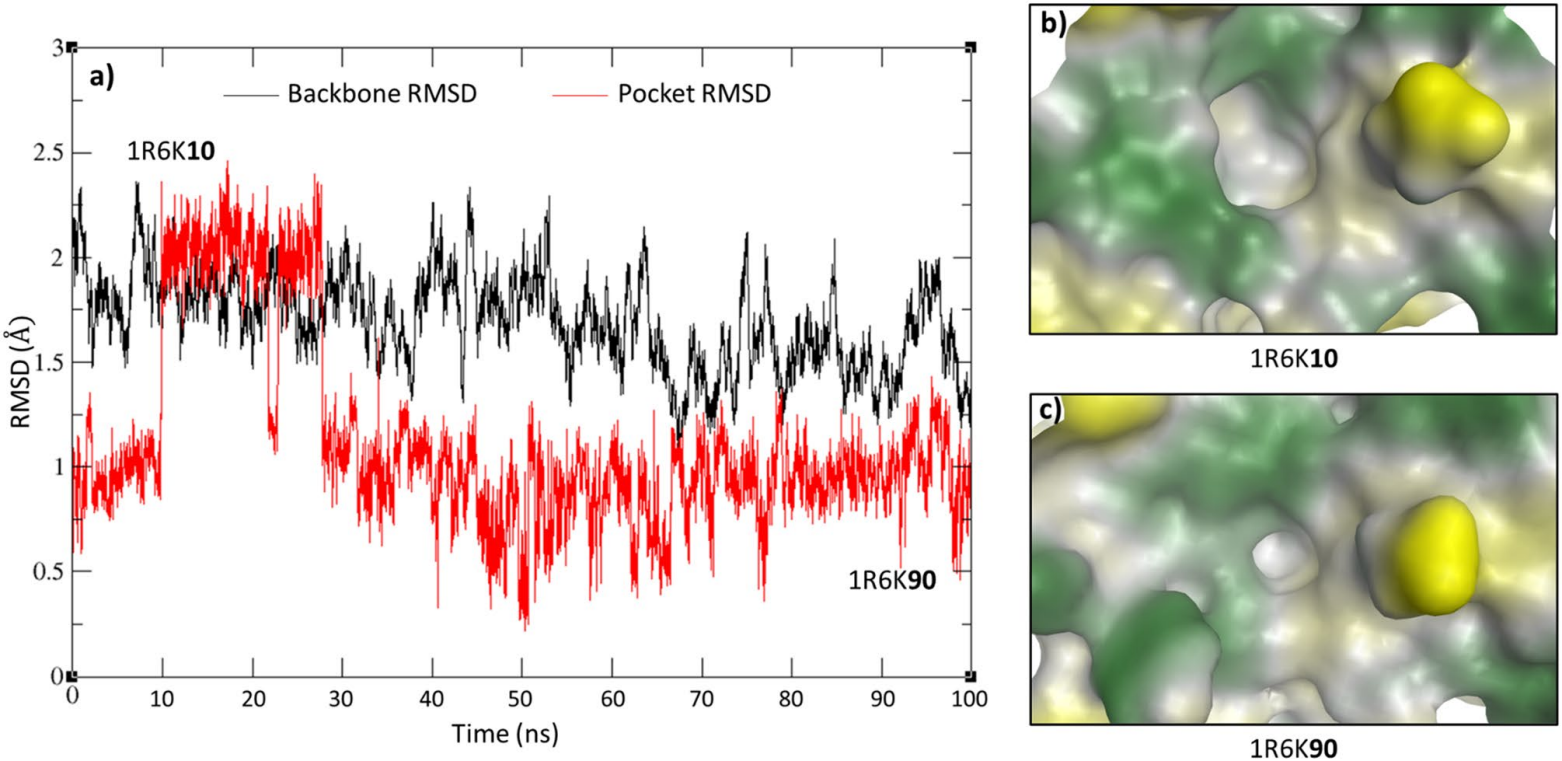
(**a**) The RMSD values of the protein backbone of HPV (PDB: 1R6K^36^) over the 100 ns MD simulation, (**b)** The HPV interface is clearly showing different topologies at the clustered structure at 10 ns compared to (**c**) the cluster at 90 ns.

In addition, we note that as an alternative to sampling multiple crystal structures, molecular dynamics simulations can be used to study protein flexibility and in particular the dynamic topology of potential protein binding sites on its surface. Therefore, as a test case, we examine the change in pocket size of apo HPV protein (PDB: 1R6K^50^) over 100 ns MD simulations (Fig. 8a). We find that the size of the binding cavity ranged from 14 to 45 spheres with an average value of 32.1 ± 9.8 spheres. As shown in Fig. 8b, the largest pocket was observed at 10 ns (n = 45 spheres), with a Dscore of 0.76; moreover, the pocket shares the same druggability classification as the only ligand-bound HPV crystal structure (n = 46 spheres; Dscore = 0.84, Table 2). The smallest pocket, on the other hand, was observed at 90 ns (Fig. 8c) and it is nearly three times smaller (n = 14 spheres) than the 10 ns-pocket. This structure showed as low Dscore as 0.45, which is far less than the Dscore values obtained by the ligand-bound and apo structures of 0.84 and 0.70 respectively. This example demonstrates how the pocket size and conformation could change substantially, influencing its druggability score and final classification (shifted from ‘druggable’ to ‘difficult’ in this case). The consequent caveat is that one may need to examine multiple crystal structures of the same target in different conformations or conduct a MD simulation where several conformations can be considered, making druggability assessments more comprehensive.

### Correlating between pocket druggability and ligand drug-likeness

It is well-known that the concept of drug-likeness applies to small drug molecules that are able to show pharmacological activity when given orally; the extent to which a biological target can bind such compounds can in turn define the extent of the protein’s druggability^14^. In recent years, a number of small organic molecule inhibitors have been reported to disrupt certain classes of PPI; nonetheless, not all of them were able to show an acceptable oral activity^4^.

A few attempts have been made to investigate the relationship between pocket druggability and ligand drug-likeness using SiteMap and QED, respectively, which provide quantitative indicators for these parameters^51^. However, in these studies, Sitescore was used to describe druggability rather than the more relevant function, Dscore. In addition, the previously studied dataset included both small molecule PPI inhibitors and antibodies, which are beyond the scope of this study. Therefore, to clarify the possible correlation between QED score and the Dscore, we examine a set of ten clinically tested oral drugs/candidates.

Up to our knowledge, these ten compounds (*Venetoclax, Navitoclax, ABT-737, GCD-0917, ASTX660, LCL161, AMG-232, CGM097, SAR405838 and RO5045337*) are the only clinically available oral PPI drugs. Drug candidates in the preclinical stages were omitted from the analysis, as the goal is to assess the progress and current status of PPI medication development beyond the initial drug discovery phase.

**Figure 9:**
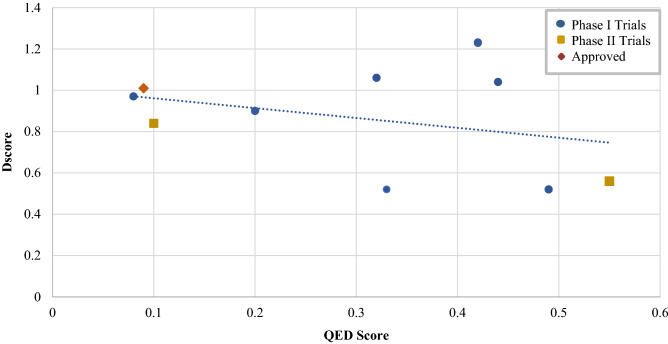
Distribution of QED scores of small molecule orally tested/approved PPI drugs relative to the druggability scores (Dscore) of identified binding site.

As shown in Fig. 9, the scatterplot indicates for a very poor correlation between the QED scores of these ligands and the Dscore values of their PPI targets (R^2^ of 0.11). This is consistent with the findings in Table 4, where a higher Dscore is not always associated with a higher number of inhibitors obeying the current drug-like rules defined by Lipinski and others^52,53^. With QED scores of less than 0.5 and multiple violations of Lipinski’s rules, nearly all these reported PPI drugs were classified as non-drug-like. The only exception here is the XIAP inhibitor ASTX660, an oral anticancer agent that is currently in phase I/II clinical trials^54^. Despite violating Lipinski’s size requirements, ASTX660 has a QED score of 0.55, indicating that it has more favorable drug-like properties than any currently approved or tested oral PPI drugs.

**Table 4:** Published PPI inhibitors classified as drug-like based on standard drug-like rules (Lipinski`s rule of 5 (Ro5) and QED scores); and relaxed PPI drug-like rules (Ro5-1).

	DCN1 (n = 8)	Bcl-xL (n = 16)	HDM2 (n = 62)	XDM2 (n = 10)	Bcl-2 (n = 14)	MDM4 (n = 8)	HPV E2 (n = 1)		Menin (n = 29)	VHL (n = 31)	IL-2 (n = 6)	XIAP (n = 31)	ZipA (n = 4)
**Standard drug-like rules (Ro5 or QED)**													
Number of drug-like inhibitors (Ro5)	6	0	7	0	0	1	0		12	20	2	14	4
Percentage of drug-like inhibitors (Ro5)	75%	0%	11.3%	0%	0%	12.5%	0%		41.4%	64.5%	33.3%	45.2%	100%
Number of drug-like inhibitors (QED)	5	0	10	3	0	2	0		13	16	1	11	3
Percentage of drug-like inhibitors (QED)	62.5%	0%	16.1%	30%	0%	25%	0%		44.8%	51.6%	16.7%	35.5%	75%
**Relaxed drug-like rules (Ro5-1)**													
Number of drug-like inhibitors (Ro5-1)	7	6	28	6	1	4	1		28	29	2	26	4
Percentage of drug-like inhibitors (Ro5-1)	87.5%	37.5%	45.2%	60%	7.1%	50%	100%		96.6%	93.5%	33.3%	82.9%	100%

### Ligand drug-likeness of PPI drugs

Recent breakthroughs in PPI Inhibition have featured a series of small molecules that modulate protein function and act as new therapeutics. However, as the mode of action and common features of these inhibitors remain unclear, it is crucial to understand the distinctive features of PPI drugs^3,55^. To do so, we need to examine published PPI drugs in terms of drug-likeness and see where they deviate from conventional drugs.

Amongst all drug-like rules, Lipinski’s rule of 5 (Ro5)^52^ is the most well-known. Moreover, the Quantitative Estimate of Drug-likeness (QED)^53^ has recently become a more extensively utilized technique for characterizing drug-likeness, particularly because it allows us to quantitatively assess and rank various compounds based on their score*.* Therefore, employing Lipinski’s Ro5^52^ and QED^53^ would provide insight into the structural features of published orthosteric PPI inhibitors, and can also describe any correlation with the druggability of their PPI targets. These two were applied together to our data set of 221 different small molecules that inhibited 12 PPI complexes from different druggability classes, all of which directly interact with the binding interface of one protein partner.

Unsurprisingly, only 30% of these inhibitors (66 out of 220) seem to follow the conventional Lipinski’s Ro5^52^ (Table 4), the remaining 154 compounds exhibited one or more violations^52^. For the QED metric, only 29% of the studied inhibitors (64 out of 220) demonstrated drug-like properties, with a QED score of 0.5 or higher. This is to be expected because QED is a more rigorous assessment tool that extends the number of considered parameters to eight^53^. Inhibitors with QED scores of less than 0.5 demonstrated unfavorable chemical properties, thereby reducing their drug-likeness. Regardless of the tool used, violations predict potential bioavailability issues; thus, as the number of violations increases, the compound is more likely to have low cell permeability and poor overall oral activity.

According to the Ro5 (Table 5), only three PPI targets have more than 50% of their respective inhibitors possessing drug-like properties, mainly violating the molecular weight and polarity parameters. It is well documented that PPI-targeting ligands violate the Ro5, due to their large size and hydrophobic nature^4,13,55–57^. Morelli et al.^55^ have studied many of them and they accordingly proposed the Rule-of-Four which expands the limits of the Ro5 to consider a higher molecular weight (Mwt > 400 Da), hydrophobicity (ALogP > 4), unsaturation index (HBA > 4) and ring complexity (Rings > 4) observed in PPI drugs compared to non-PPI drugs. For instance, HDM2 inhibitor ABT-737 exhibited a very high oral bioavailability in phase II trials despite being large in size (Mwt = 813.43 Da)^13,58^. This is because PPI inhibitors may bind to multiple high energy hots spots rather than binding to a well-defined pocket, which requires them to have special characteristics in order to do so^14,59^.

**Table 5:** Calculated physiochemical properties of published inhibitors based on Lipinski’s rule of 5 (Ro5) and QED scores. Data represents mean ± standard deviation.

	Lipinski’s (Ro5)				QED
	HBA	HBD	LogP	Molecular weight	
DCN-1	2.63 ± 1.30	2.00 ± 0.95	4.00 ± 1.33	469.92 ± 62.34	0.54 ± 0.16
Bcl-xL	3.76 ± 1.52	1.52 ± 0.8	6.21 ± 1.65	632.03 ± 165.62	0.21 ± 0.20
HDM2	3.35 ± 1.66	1.11 ± 1	5.55 ± 1.28	549.59 ± 100.41	0.37 ± 0.14
XDM2	3.20 ± 1.69	1.4 ± 1.43	1.34 ± 6.25	537.70 ± 114.85	0.58 ± 0.19
Bcl-2	5.46 ± 1.51	1.85 ± 0.99	5.64 ± 1.57	847.86 ± 132.18	0.14 ± 0.10
MDMX	3.25 ± 1.98	1.25 ± 0.89	5.49 ± 1.32	568.20 ± 147.03	0.37 ± 0.24
HPV E2	6.00	1.00	6.25	607.44	0.23
Menin	3.64 ± 1.28	1.04 ± 1.04	4.40 ± 1.54	501.04 ± 103.45	0.45 ± 0.22
VHL	5.29 ± 1.55	2.97 ± 0.91	1.66 ± 1.45	486.37 ± 138.57	0.51 ± 0.21
IL-2	3.33 ± 1.63	1.67 ± 1.02	2.72 ± 0.93	495.51 ± 208.22	0.22 ± 0.20
XIAP	3.60 ± 2.67	1.39 ± 1.65	2.97 ± 1.51	477.98 ± 215.31	0.44 ± 0.20
ZipA	3 ± 1.41	2.25 ± 2.06	2.70 ± 0.41	353.80 ± 83.76	0.62 ± 0.14

The rule of four serves as a descriptive tool that describes the overall physicochemical properties of currently known PPI inhibitors*.* Hence, it becomes more clear that PPI drugs tend to be larger in size, more hydrophobic and contain a large number of aromatic rings^55,60^. Alternately, we here propose relaxing Lipinski’s parameters by allowing one violation within the Ro5^52^ and we called it “Ro5-1”. This rule has been optimized to allow for some deviation from conventional drug-like molecules, and validated by PPI inhibitors showing oral bioavailability in clinical settings. According to the relaxed drug-like rules (Ro5-1), 142 of published PPI inhibitors are drug-like, which is more than double the number of inhibitors suggested by standard drug-like rules (Ro5 and QED). This would greatly expand the chemical space for PPI targets, allowing for more successful hits with favourable ADMET properties.

**Figure 10:**
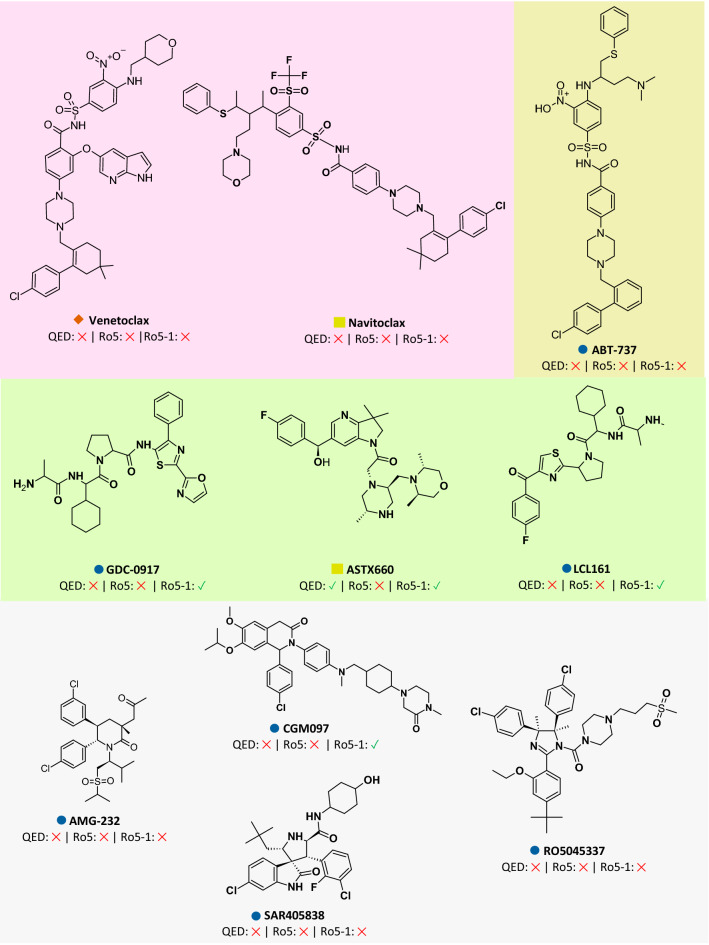
A subset of orally tested/approved PPI drugs assessed using standard drug-like rules (Lipinski’s and QED), and relaxed PPI drug-like rules (Ro5-1). Note: **✓** for Drug-like; ✕ for nondrug-like.

Applying the Ro5-1 to a set of ten clinically tested oral drugs/candidates, shown in Fig. 10, would further confirm this, as all listed PPI drugs violate Lipinski’s Ro5 and only one (ASTX-600) has a QED score in the drug-like range. In contrast, the Ro5-1 would classify 4 out of the 10 PPI drugs as drug-like, 40% higher than the conventional Lipinski’s rules. Taking this into account, it is clear that poor chemical-biological candidates can have promising therapeutic properties and that such compounds should not be dismissed due to their low likelihood of being developed as marketed drugs^55^.

To summarize, it seems that the current drug-like rules do not necessarily apply on the PPI inhibitors as no correlation was found between the ligand drug-likeness and the pocket druggability parameters. This informs researchers about the importance of proposing a PPI-specific drug-likeness rules similar to what has been suggested in this study for PPI interface druggability assessment and classification.

## Conclusion

In this study, we assessed the druggability of 12 commonly targeted PPIs using SiteMap, revealing a range of druggability scores to their respective binding sites. We attribute these differences to their unique structural and physiochemical features. Interestingly, these features were used to propose a new druggability classification system geared towards PPI targets. The newly suggested system classifies PPIs into four categories based on their druggability score (Dscore) as well as the inhibition activity of their respective inhibitors. The analysis of the different Dscore parameters shows that the pocket size and hydrophilicity have the strongest correlation with Dscore values of the studied PPIs. This is readily detected in the moderately druggable proteins, for which binding pockets are small in size and hydrophilic in nature, such as IL-2 and XIAP. We also studied the importance of protein flexibility on calculated druggability score, and found that protein conformational changes accompanying ligand binding in proteins such as Bcl-xL and IL-2 resulted in significantly higher druggability scores and more favorable structural features than observed for the apo form or, to a lesser extent, the protein/peptide bound structures MD simulations were in favor of this too as it showed significant variations in the pocket size and Dscore values throughout the course of the simulations. Finally, published PPI inhibitors were studied to determine their “drug-likeness” using Lipinski’s rule of 5 (Ro5) and QED score. Our findings revealed that the vast majority of PPI drugs, including those that have been orally tested/marketed, exceeded the typically acceptable size and hydrophobicity parameters. This made us propose relaxing the drug-like rules and allow at least on violation (Ro5-1) which had a considerable impact on preventing the exclusion of many important PPI drug candidates that have already shown clinical value and could have been eliminated by the conventional drug-like rules. This work proposes a PPI-specific classification scheme that will assist researchers in assessing druggability and identifying PPI inhibitors with a potential oral activity.

## Methods

### Selection of a dataset of protein–protein interaction targets

The Wells^28^ set and the 2P2I database^61^ are commonly used data sets for the validation of in silico PPI assessment tools. Accordingly, 12 protein–protein interaction targets have been derived from both lists and included in our PPI dataset: these proteins are Defective in cullin neddylation protein 1 (DNC1), Menin, Human double minute 2 (HDM2), Xenopus double minute 2 (XDM2), Protein MDM4 (MDMX), Interleukin-2 (IL-2), Regulatory protein E2 (HPV E2), Bcl2-associated agonist of cell death (Bcl-2), Apoptosis regulator Bc-X (Bcl-xL), Von Hippel Lindau protein (VHL), E3 ubiquitin-protein ligase XIAP (XIAP) and Cell division protein ZipA (ZipA). A search was then conducted on the protein data bank (PDB)^62^ to obtain all ligand-bound, protein/peptide bound and apo structures for each PPI from the aforementioned set. PPI structures containing a covalent inhibitor or an inhibitor not bound to the PPI interface, or with mutated residues, were excluded from the PPI dataset. Overall, a total of 320 crystal structures were included in the study.

### Preparation of protein–protein interaction crystal structures

Each crystal structure had solvent atoms and co-crystalized heteroatoms removed using Molecular Operating Environment (MOE)^23^. If multiple chains were present for the same protein, they were manually removed so that only the chain bound to the inhibitor remained. After that, all structures were corrected to add missing atoms, residues, chains or loops. Protonation states were assigned to each atom using Protonate3D in MOE. These protein crystal structures were then imported into Maestro^63^ to ensure the structural correctness of prepared structures, proteins were refined using the Protein Preparation wizard^64^ module where hydrogens were added through hydrogen bond optimization and subsequently underwent restrained minimized to a lower energy state with a maximum RMSD of 0.30 Å.

### Sequence alignment and structural superposition of inhibitor-bound protein with apo and protein/peptide bound structures

For protein/peptide bound complexes, secondary structure assignments were manually removed from each complex. Given both apo and protein/peptide complexes contained one chain of the desired protein, they underwent sequence alignment and structural superimposition with an inhibitor-bound protein from the same family, using the align/superimpose feature in MOE^23^. Only the inhibitor-bound protein was then removed, keeping its bound inhibitor in the PPI pocket of the apo structure or the protein/peptide bound complexes. Consequently, the aligned inhibitor was used to identify the respective pocket in the PPI interface.

### Druggability assessment of PPI interface using sitemap

Proteins were then processed through the SiteMap^20^ module with all settings kept to default. To define protein binding pockets, the “Evaluate single binding region” option was selected. SiteMap generates various physiochemical descriptors including size, volume, and degree of enclosure, hydrophobicity and hydrophilicity. Most importantly, it scores a protein binding pocket by calculating its Dscore:

$${\text{Dscore }} = 0.094\sqrt {\text{n}} + 0.60{\text{e}} - 0.{\text{324p,}}$$

where n is the number of site points, e is the enclosure factor and p is the hydrophilic factor.

### Molecular dynamics simulations

Molecular dynamics (MD) simulation studies were conducted to investigate the dynamic nature of protein by generating numerous conformations of the protein for druggability assessment. The AMBER18 package^65^ was used to run MD simulations of the apo form of HPV protein (PDB code 1R6K^50^). Partial charges and other parameters were assigned to the protein structure using the ff19SB force field. The protein system was built using the xleap module of AmberTools, where it was neutralized by the addition of Na + counter ions and solvated by a truncated octahedral box of TIP3P water. The energy of the system was subsequently minimized in two steps using the pmemd program in the AMBER 18 package^65^ first, all solute atoms were constrained with a force constant of 500 kcal mol^−1^ (− 2) during minimization, and then the entire system was subjected to minimization without any constraints. The system was then gradually heated from 0 to 300 K in the NVT ensemble. Using the Langevin thermostat, the SHAKE algorithm was applied to all bonds containing hydrogen atoms with a collision frequency of ps-1^66^. Finally, the protein was subjected to a 100 ns MD simulation in the NPT ensemble, with the system temperature and pressure set to 300 K and 1.01 × 105 Pa, respectively.

### Clustering analysis of MD trajectories

After completion of MD runs, trajectories were analyzed using DBSCAN^67^ via the cpptraj module of AmberTools^68^. Every tenth frame (10 ns) was used in clustering. Ions and solvent molecules were removed from each system. The distance cutoff between points for forming a cluster, *ε*, was set to 3.0 (default value). The trajectory files were evaluated by extracting the graph of root-mean-square deviation (RMSD) using centering utilities. The size of the binding site on the protein interface was computed for each structure using the Sitemap^20^ module of the Schrodinger’s Maestro^63^.

### Assessing the drug-likeness of PPI-targeting ligands using the Ro5 and QED

To evaluate the drug-likeness of published PPI inhibitors, these ligands were isolated from their protein complexes and compiled into databases. Recent review articles were used to obtain orally tested/approved PPI drugs and their respective structures^3,51^. Their structural features were then studied using Lipinski’s rule of 5 (Ro5)^52^ and the Quantitative Estimate of Drug-likeness (QED)^53^. Individual physiochemical parameters were calculated for each inhibitor, including the number of hydrogen bond donors (HBD), number of hydrogen bond acceptors (HBA), molecular weight (Mwt) and LogP to estimate their drug-likeness in accordance with the Ro5^52^. Ligands were then reprocessed using QED^53^ which classifies and ranks ligands according to their QED score. The QED score takes into account 8 parameters: molecular weight (Mwt), number of rotatable bonds (nRotB), number hydrogen bond donors (HBD), number of hydrogen bond acceptors (HBA), octanol–water partition coefficient (ALogP), number of aromatic rings (Arom), number of structural alerts (Alerts) and molecular polar surface area (PSA). The QED score ranges from 0 to 1; a QED score of 0 indicates unfavorable non-drug-like properties, whereas a score of 1 indicates favorable drug-like properties.

MOE^23^ descriptors Lip_drug-like and Lip_violations were calculated for each inhibitor to estimate their drug-likeness using the relaxed drug-like rules (Ro5-1). A drug-like inhibitor is expected to have a Lip_drug-like score of 1 if it has no more than one violation. Inhibitors with more than one violation will have a Lip_drug-like score of 0 and therefore are classified as non-drug-like.

## Supplementary Information

Supplementary Information 1.

Supplementary Information 2.

Supplementary Information 3.

Supplementary Information 4.

## Data Availability

All data generated or analysed during this study are included in this published article (and its supplementary information files). Requests for material should be made to the corresponding authors.
