# Supplementary material for: Structure-based assessment and druggability classification of protein–protein interaction sites
Source: Sci Rep. 2022 May 13;12:7975. doi: 10.1038/s41598-022-12105-8 (PMC9106675; doi:10.1038/s41598-022-12105-8)

# Supporting Information

# Structure-based assessment and druggability classification of protein-protein interaction sites

# *Lara Alzyoud1,2, Richard A. Bryce3, Mohammad Al Sorkhy4, Noor Atatreh1,2, and Mohammad A. Ghattas1,2 **

1College of Pharmacy, Al Ain University, Abu Dhabi, United Arab Emirates, 64141

2 AAU Health and Biomedical Research Center, Al Ain University, Abu Dhabi, United Arab Emirates, 64141

3Division of Pharmacy and Optometry, School of Health Sciences, University of Manchester, Oxford Road, Manchester M13 9PL, UK

4Department of Biology, University of Toronto, Ontario, Canada

* [mohammad.ghattas@aau.ac.ae](mailto:mohammad.ghattas@aau.ac.ae)

**Content:**

**Table S1.** Ligand-bound protein crystal structures included in the PPI dataset.

**Table S2.** Apo protein crystal structures included in the PPI dataset.

**Table S3.** Protein/Peptide-bound protein crystal structures included in the PPI dataset.

**Table S4.** Output from SiteMap for all 320 protein crystal structures belonging to 12 PPI targets.

**Table S5.** Reference ligand-bound PDB that was superimposed into Apo and Protein/Peptide- bound structures.

**Table S6.** Comparison of the binding affinity values across 12 PPI targets.

**Figure S1.** Linear regression plots of median Dscore against median pocket size (n), median enclosure factor (e), and median hydrophilicity factor (p).

**Table S1.** Ligand-bound protein crystal structures included in the PPI dataset.

| **Protein** | **Structure Type** | ***PDB*** | ***Resolution*** | ***Ligand*** |
| --- | --- | --- | --- | --- |
| DCN1 | Ligand-bound | 5UFI | 2.58 | 8B1 |
| DCN1 | Ligand-bound | 5V83 | 2.00 | 8Z7 |
| DCN1 | Ligand-bound | 5V86 | 1.37 | 8ZA |
| DCN1 | Ligand-bound | 5V88 | 1.60 | 8ZD |
| DCN1 | Ligand-bound | 6BG3 | 1.05 | DOJ |
| DCN1 | Ligand-bound | 6BG5 | 1.10 | DQD |
| DCN1 | Ligand-bound | 6P5V | 1.40 | O37 |
| DCN1 | Ligand-bound | 6P5W | 1.69 | O0A |
| Bcl-xL | Ligand-bound | 2YXJ | 2.20 | N3C |
| Bcl-xL | Ligand-bound | 6VWC | 1.60 | RQ7 |
| Bcl-xL | Ligand-bound | 7JGV | 2.05 | V9S |
| Bcl-xL | Ligand-bound | 7JGW | 1.30 | V9S |
| Bcl-xL | Ligand-bound | 3ZK6 | 2.48 | H1I |
| Bcl-xL | Ligand-bound | 3ZLN | 2.29 | H0Y |
| Bcl-xL | Ligand-bound | 3ZLO | 2.60 | X8U |
| Bcl-xL | Ligand-bound | 3ZLR | 2.03 | X0B |
| Bcl-xL | Ligand-bound | 4C5D | 2.30 | X0R |
| Bcl-xL | Ligand-bound | 4C52 | 2.05 | X0D |
| Bcl-xL | Ligand-bound | 3QKD | 2.02 | HI0 |
| Bcl-xL | Ligand-bound | 4TUH | 1.80 | 38H |
| Bcl-xL | Ligand-bound | 4QVX | 2.10 | 3CQ |
| Bcl-xL | Ligand-bound | 4EHR | 2.09 | 0Q5 |
| Bcl-xL | Ligand-bound | 3SPF | 1.70 | B50 |
| Bcl-xL | Ligand-bound | 37P7 | 1.40 | 03B |
| HDM2 | Ligand-bound | 3JZK | 2.10 | YIN |
| HDM2 | Ligand-bound | 3W69 | 1.90 | LTZ |
| HDM2 | Ligand-bound | 3VZV | 2.80 | VZV |
| HDM2 | Ligand-bound | 4HBM | 1.90 | 0Y7 |
| HDM2 | Ligand-bound | 4JV9 | 2.20 | 1MN |
| HDM2 | Ligand-bound | 4JV7 | 2.50 | 1MO |
| HDM2 | Ligand-bound | 4JVR | 1.70 | 1MT |
| HDM2 | Ligand-bound | 4JVE | 2.30 | 1MQ |
| HDM2 | Ligand-bound | 4JWR | 2.35 | 1MY |
| HDM2 | Ligand-bound | 4DIJ | 1.90 | BLF |
| HDM2 | Ligand-bound | 4HG7 | 1.60 | NUT |
| HDM2 | Ligand-bound | 4MDN | 1.90 | Y30 |
| HDM2 | Ligand-bound | 4MDQ | 2.12 | 28W |
| HDM2 | Ligand-bound | 4ERE | 1.80 | 0R2 |
| HDM2 | Ligand-bound | 4ERF | 2.00 | 0R3 |
| HDM2 | Ligand-bound | 4OBA | 1.60 | 2TW |
| HDM2 | Ligand-bound | 4OAS | 1.70 | 2SW |
| HDM2 | Ligand-bound | 4OCC | 1.80 | 2TZ |
| HDM2 | Ligand-bound | 4ODE | 1.80 | 2U0 |
| HDM2 | Ligand-bound | 4ODF | 2.20 | 2U1 |
| HDM2 | Ligand-bound | 4OGT | 1.54 | 2U6 |
| HDM2 | Ligand-bound | 4OGV | 2.20 | 2U7 |
| HDM2 | Ligand-bound | 4OGN | 1.38 | 2U5 |
| HDM2 | Ligand-bound | 4OQ3 | 2.30 | 2V8 |
| HDM2 | Ligand-bound | 4QOC | 1.70 | 35T |
| HDM2 | Ligand-bound | 1RV1 | 2.30 | 1MZ |
| HDM2 | Ligand-bound | 1T4E | 2.60 | DIZ |
| HDM2 | Ligand-bound | 4QO4 | 1.70 | 35S |
| HDM2 | Ligand-bound | 7BIV | 1.64 | TUW |
| HDM2 | Ligand-bound | 7BIT | 2.13 | TV5 |
| HDM2 | Ligand-bound | 7BJ6 | 1.59 | TVK |
| HDM2 | Ligand-bound | 7BIR | 2.02 | TUZ |
| HDM2 | Ligand-bound | 7BMG | 1.83 | U3Z |
| HDM2 | Ligand-bound | 6Q96 | 1.80 | HRE |
| HDM2 | Ligand-bound | 6Q9O | 1.21 | HU8 |
| HDM2 | Ligand-bound | 6Q9H | 2.00 | HRH |
| HDM2 | Ligand-bound | 6Q9L | 1.13 | HTZ |
| HDM2 | Ligand-bound | 3LBL | 1.60 | MI6 |
| HDM2 | Ligand-bound | 3LBK | 2.30 | K23 |
| HDM2 | Ligand-bound | 4WT2 | 1.42 | 3UD |
| HDM2 | Ligand-bound | 5C5A | 1.15 | NUT |
| HDM2 | Ligand-bound | 5Z02 | 1.35 | NUT |
| HDM2 | Ligand-bound | 5TRF | 2.10 | 7HC |
| HDM2 | Ligand-bound | 6GGN | 2.00 | EYH |
| HDM2 | Ligand-bound | 5OAI | 2.00 | B5K |
| HDM2 | Ligand-bound | 5OC8 | 1.56 | 9QW |
| HDM2 | Ligand-bound | 5LN2 | 1.58 | 6ZT |
| HDM2 | Ligand-bound | 5J7G | 1.85 | 6GG |
| HDM2 | Ligand-bound | 5J7F | 2.00 | 6GG |
| HDM2 | Ligand-bound | 5LAZ | 1.66 | 6ST |
| HDM2 | Ligand-bound | 5LAW | 1.64 | 6SJ |
| HDM2 | Ligand-bound | 5LAV | 1.73 | 6SK |
| HDM2 | Ligand-bound | 5LAY | 2.71 | 6SS |
| HDM2 | Ligand-bound | 5HMK | 2.17 | 62Q |
| HDM2 | Ligand-bound | 5HMI | 1.74 | 62T |
| HDM2 | Ligand-bound | 5HMH | 1.79 | 62R |
| HDM2 | Ligand-bound | 4ZFI | 2.00 | 4NJ |
| HDM2 | Ligand-bound | 4ZGK | 2.00 | 4NX |
| HDM2 | Ligand-bound | 4ZYC | 1.95 | 4SS |
| HDM2 | Ligand-bound | 4ZYF | 1.80 | 4T4 |
| HDM2 | Ligand-bound | 4ZYI | 1.67 | 4TH |
| HDM2 | Ligand-bound | 5ZXF | 1.25 | NUT |
| HDM2 | Ligand-bound | 6I3S | 1.77 | H28 |
| HDM2 | Ligand-bound | 3VBG | 2.80 | 03M |
| XDM2 | Ligand-bound | 4IPF | 1.70 | 1F0 |
| XDM2 | Ligand-bound | 4J3E | 1.91 | NUT |
| XDM2 | Ligand-bound | 4J74 | 1.20 | I18 |
| XDM2 | Ligand-bound | 4JRG | 1.90 | I09 |
| XDM2 | Ligand-bound | 4JSC | 2.50 | 1OY |
| XDM2 | Ligand-bound | 4J7D | 1.25 | I31 |
| XDM2 | Ligand-bound | 4J7E | 1.63 | I29 |
| XDM2 | Ligand-bound | 4LWT | 1.60 | 20Q |
| XDM2 | Ligand-bound | 4LWU | 1.14 | 20U |
| XDM2 | Ligand-bound | 4LWV | 2.32 | 20W |
| Bcl-2 | Ligand-bound | 6QGH | 2.00 | 1XJ |
| Bcl-2 | Ligand-bound | 6QGK | 1.80 | J1Q |
| Bcl-2 | Ligand-bound | 6QGJ | 1.90 | J1T |
| Bcl-2 | Ligand-bound | 7LHB | 2.07 | XZD |
| Bcl-2 | Ligand-bound | 4IEH | 2.10 | 1E9 |
| Bcl-2 | Ligand-bound | 2W3L | 2.10 | DRO |
| Bcl-2 | Ligand-bound | 4LVT | 2.05 | 1XJ |
| Bcl-2 | Ligand-bound | 4LXD | 1.90 | 1XV |
| Bcl-2 | Ligand-bound | 4MAN | 2.07 | 1Y1 |
| Bcl-2 | Ligand-bound | 6GL8 | 1.40 | F3Q |
| Bcl-2 | Ligand-bound | 6O0P | 1.80 | LBM |
| Bcl-2 | Ligand-bound | 6O0O | 2.00 | F3Q |
| Bcl-2 | Ligand-bound | 6O0L | 2.20 | LBM |
| Bcl-2 | Ligand-bound | 6O0K | 1.62 | LBM |
| Bcl-2 | Ligand-bound | 6O0M | 1.75 | LBM |
| Bcl-2 | Ligand-bound | 4AQ3 | 2.40 | 398 |
| Bcl-2 | Ligand-bound | 6QGG | 1.50 | J1H |
| MDMX | Ligand-bound | 7C3Y | 1.63 | NUT |
| MDMX | Ligand-bound | 7C3Q | 1.80 | NUT |
| MDMX | Ligand-bound | 7C44 | 1.65 | NUT |
| MDMX | Ligand-bound | 6Q9W | 1.55 | HRT |
| MDMX | Ligand-bound | 6Q9Y | 1.20 | HRQ |
| MDMX | Ligand-bound | 6Q9Q | 2,10 | HUE |
| MDMX | Ligand-bound | 6Q9S | 2.40 | HRN |
| MDMX | Ligand-bound | 6Q9U | 2.40 | HRE |
| MDMX | Ligand-bound | 3LBJ | 1.50 | WW8 |
| MDMX | Ligand-bound | 3U15 | 1.80 | 03M |
| HPV E2 | Ligand-bound | 1R6N | 2.40 | 434 |
| Menin | Ligand-bound | 4GQ3 | 1.56 | 0RO |
| Menin | Ligand-bound | 4GQ4 | 1.27 | 0RT |
| Menin | Ligand-bound | 4X5Y | 1.59 | 3XW |
| Menin | Ligand-bound | 4X5Z | 1.86 | 6E6 |
| Menin | Ligand-bound | 4OG3 | 2.01 | 2S6 |
| Menin | Ligand-bound | 4OG4 | 1.45 | 2VK |
| Menin | Ligand-bound | 4OG5 | 1.63 | 2S7 |
| Menin | Ligand-bound | 4OG6 | 1.49 | 2S9 |
| Menin | Ligand-bound | 4OG7 | 2.08 | 2SE |
| Menin | Ligand-bound | 4OG8 | 1.53 | 2SF |
| Menin | Ligand-bound | 6O5I | 3.10 | LMY |
| Menin | Ligand-bound | 6S2K | 3.10 | KTQ |
| Menin | Ligand-bound | 6PKC | 1.90 | OP4 |
| Menin | Ligand-bound | 6EA1 | 3.10 | 7LH |
| Menin | Ligand-bound | 6B41 | 2.61 | CJV |
| Menin | Ligand-bound | 5DB0 | 1.50 | 58P |
| Menin | Ligand-bound | 5DB1 | 1.86 | 58O |
| Menin | Ligand-bound | 5DB2 | 1.54 | 58R |
| Menin | Ligand-bound | 5DB3 | 1.71 | 58Q |
| Menin | Ligand-bound | 5DDF | 1.66 | 5A1 |
| Menin | Ligand-bound | 5DDE | 1.78 | 5A0 |
| Menin | Ligand-bound | 5DDA | 1.83 | 59M |
| Menin | Ligand-bound | 5DDB | 1.54 | 59Q |
| Menin | Ligand-bound | 5DDC | 1.62 | 59V |
| Menin | Ligand-bound | 5DDD | 2.14 | 59X |
| Menin | Ligand-bound | 5DD9 | 1.62 | 59K |
| Menin | Ligand-bound | 6BXH | 2.44 | EE7 |
| Menin | Ligand-bound | 6BY8 | 1.90 | FNV |
| Menin | Ligand-bound | 6BXY | 1.82 | EEV |
| VHL | Ligand-bound | 3ZRC | 2.90 | L8B |
| VHL | Ligand-bound | 3ZTC | 2.65 | TR0 |
| VHL | Ligand-bound | 3ZTD | 2.79 | ZTD |
| VHL | Ligand-bound | 3ZUN | 2.50 | ZUN |
| VHL | Ligand-bound | 4B9K | 2.00 | TG0 |
| VHL | Ligand-bound | 4B95 | 2.80 | UCK |
| VHL | Ligand-bound | 4BKS | 2.20 | X6C |
| VHL | Ligand-bound | 4BKT | 2.35 | QD0 |
| VHL | Ligand-bound | 4W9C | 2.20 | 3JG |
| VHL | Ligand-bound | 4W9D | 2.20 | 3JK |
| VHL | Ligand-bound | 4W9E | 2.60 | 3JT |
| VHL | Ligand-bound | 4W9F | 2.10 | 3JU |
| VHL | Ligand-bound | 4W9G | 2.70 | 3JV |
| VHL | Ligand-bound | 4W9H | 2.10 | 3JF |
| VHL | Ligand-bound | 4W9I | 2.40 | 3JS |
| VHL | Ligand-bound | 4W9J | 2.20 | 3JH |
| VHL | Ligand-bound | 4W9K | 2.10 | 3JO |
| VHL | Ligand-bound | 4W9L | 2.20 | 3JJ |
| VHL | Ligand-bound | 5LLI | 2.40 | 6Z3 |
| VHL | Ligand-bound | 5NVV | 2.10 | 9BT |
| VHL | Ligand-bound | 5NVW | 2.20 | 9BW |
| VHL | Ligand-bound | 5NVY | 2.90 | 9B5 |
| VHL | Ligand-bound | 5NVZ | 2.70 | 9BN |
| VHL | Ligand-bound | 5NW0 | 2.30 | 9BK |
| VHL | Ligand-bound | 5NW1 | 2.10 | 9BH |
| VHL | Ligand-bound | 5NW2 | 2.20 | 9B8 |
| VHL | Ligand-bound | 6FMI | 2.80 | DV2 |
| VHL | Ligand-bound | 6FMJ | 2.45 | DV5 |
| VHL | Ligand-bound | 6FMK | 2.75 | DV8 |
| VHL | Ligand-bound | 6HAY | 2.24 | FX8 |
| VHL | Ligand-bound | 6HR2 | 1.76 | FWZ |
| IL-2 | Ligand-bound | 1PY2 | 2.80 | FRH |
| IL-2 | Ligand-bound | 1PW6 | 2.60 | FRB |
| IL-2 | Ligand-bound | 1QVN | 2.70 | FRI |
| IL-2 | Ligand-bound | 1M49 | 2.00 | CMM |
| IL-2 | Ligand-bound | 1M48 | 1.95 | FRG |
| IL-2 | Ligand-bound | 4NEJ | 1.92 | 2K1 |
| XIAP | Ligand-bound | 4KJU | 1.60 | 1RH |
| XIAP | Ligand-bound | 4KJV | 1.70 | 1RK |
| XIAP | Ligand-bound | 4KMP | 1.95 | GT6 |
| XIAP | Ligand-bound | 4HY0 | 2.84 | 1AQ |
| XIAP | Ligand-bound | 6H6Q | 2.63 | FUK |
| XIAP | Ligand-bound | 6H6R | 2.03 | FUE |
| XIAP | Ligand-bound | 5C0K | 2.20 | 4WK |
| XIAP | Ligand-bound | 5C0L | 2.60 | 4WJ |
| XIAP | Ligand-bound | 5C3H | 2.65 | 4XE |
| XIAP | Ligand-bound | 5C3K | 2.02 | 4XF |
| XIAP | Ligand-bound | 5C7B | 2.68 | 4YD |
| XIAP | Ligand-bound | 5C7A | 2.36 | 4YE |
| XIAP | Ligand-bound | 5C7D | 2.25 | 4YF |
| XIAP | Ligand-bound | 5C7C | 2.32 | 4YC |
| XIAP | Ligand-bound | 5C83 | 2.33 | 4YN |
| XIAP | Ligand-bound | 5C84 | 2.36 | 4YL |
| XIAP | Ligand-bound | 5M6F | 2.39 | 7HU |
| XIAP | Ligand-bound | 5M6H | 2.50 | 7J6 |
| XIAP | Ligand-bound | 6EY2 | 2.70 | C3T |
| XIAP | Ligand-bound | 5OQW | 2.31 | A4E |
| XIAP | Ligand-bound | 5M6E | 2.32 | 7HT |
| XIAP | Ligand-bound | 5M6L | 2.61 | 7H9 |
| XIAP | Ligand-bound | 5M6M | 2.37 | 7H8 |
| XIAP | Ligand-bound | 3HL5 | 1.80 | 9JZ |
| XIAP | Ligand-bound | 3EYL | 3.00 | SMK |
| XIAP | Ligand-bound | 4EC4 | 3.30 | 0O6 |
| XIAP | Ligand-bound | 3G76 | 3.00 | CZ3 |
| XIAP | Ligand-bound | 3CM2 | 2.50 | X23 |
| XIAP | Ligand-bound | 3CLX | 2.70 | X22 |
| XIAP | Ligand-bound | 3CM7 | 3.10 | X22 |
| XIAP | Ligand-bound | 2JK7 | 2.82 | BI6 |
| XIAP | Ligand-bound | 2OPY | 2.80 | CO9 |
| Zip-A | Ligand-bound | 1SIJ | 2.18 | IQZ |
| Zip-A | Ligand-bound | 1S1S | 2.10 | WAC |
| Zip-A | Ligand-bound | 1Y2F | 2.00 | WAI |
| Zip-A | Ligand-bound | 1Y2G | 1.90 | CL3 |

**Table S2.** Apo protein crystal structures included in the PPI dataset.

| **Protein** | **Structure Type** | ***PDB*** | ***Resolution*** |
| --- | --- | --- | --- |
| Bcl-xL | Apo | 3CVA | 2.70 |
| Bcl-xL | Apo | 1R2D | 1.95 |
| Bcl-xL | Apo | 1R2E | 2.10 |
| Bcl-xL | Apo | 1R2I | 2.00 |
| HDM2 | Apo | 3VB3 | 2.20 |
| HPV E2 | Apo | 1R6K | 2.50 |
| Menin | Apo | 3U84 | 2.50 |
| IL-2 | Apo | 3QB1 | 3.10 |
| IL-2 | Apo | 3INK | 2.50 |
| IL-2 | Apo | 1M47 | 1.99 |
| IL-2 | Apo | 1M4C | 2.40 |
| XIAP | Apo | 4J3Y | 1.45 |
| XIAP | Apo | 2POI | 1.80 |
| Zip-A | Apo | 1F46 | 1.50 |

**Table S3.** Protein/Peptide-bound protein crystal structures included in the PPI dataset

| **Protein** | **Structure Type** | ***PDB*** | ***Resolution*** | ***Protein/Peptide*** |
| --- | --- | --- | --- | --- |
| DCN1 | Peptide/Protein bound | 3TDU | 1.50 | Cullin-1 and UBC12 |
| DCN1 | Peptide/Protein bound | 3TDZ | 1.50 | Cullin-1 and NEDD8-conjugating enzyme Ubc12 |
| Bcl-xL | Peptide/Protein bound | 2P1L | 2.50 | Beclin-1 |
| Bcl-xL | Peptide/Protein bound | 1PQ1 | 1.65 | Bim |
| Bcl-xL | Peptide/Protein bound | 2BZW | 2.30 | BAD |
| Bcl-xL | Peptide/Protein bound | 3FDL | 1.78 | Bim BH3 peptide |
| HDM2 | Peptide/Protein bound | 5AFG | 1.90 | Stapled peptide |
| HDM2 | Peptide/Protein bound | 4UMN | 1.99 | M06 |
| HDM2 | Peptide/Protein bound | 3TPX | 1.80 | D-peptide inhibitor |
| HDM2 | Peptide/Protein bound | 3JZR | 2.10 | pDI6W peptide |
| HDM2 | Peptide/Protein bound | 6H22 | 2.01 | Stapled peptide |
| HDM2 | Peptide/Protein bound | 6T2E | 2.40 | Stapled peptide |
| HDM2 | Peptide/Protein bound | 6T2D | 1.80 | Stapled peptide |
| HDM2 | Peptide/Protein bound | 5XXK | 1.66 | Hydrocarbon stapled petide |
| HDM2 | Peptide/Protein bound | 6AAW | 2.00 | D amino acid containing stapled peptide |
| HDM2 | Peptide/Protein bound | 6KZU | 1.79 | D-linear peptide |
| HDM2 | Peptide/Protein bound | 7AD0 | 2.07 | Modified p53 peptide |
| HDM2 | Peptide/Protein bound | 5VK0 | 1.80 | 12-mer Stapled peptide inhibitor PMI |
| HDM2 | Peptide/Protein bound | 5UMM | 1.65 | Peptide inhibitor M3 |
| HDM2 | Peptide/Protein bound | 2GV2 | 1.80 | 8-mer P53 peptide analogue |
| HDM2 | Peptide/Protein bound | 6Y4Q | 1.63 | Stapled peptide |
| HDM2 | Peptide/Protein bound | 2AXI | 1.40 | Cyclic 8-mer peptide |
| HDM2 | Peptide/Protein bound | 4UE1 | 1.45 | YS-01 |
| HDM2 | Peptide/Protein bound | 4UD7 | 1.60 | YS-02 |
| HDM2 | Peptide/Protein bound | 4HFZ | 2.69 | P53 |
| HDM2 | Peptide/Protein bound | 6HFA | 1.79 | LM266 |
| HDM2 | Peptide/Protein bound | 6IM9 | 3.30 | CueO-PM2 sensor |
| HDM2 | Peptide/Protein bound | 1YCR | 2.60 | P53 |
| XDM2 | Peptide/Protein bound | 1YCQ | 2.30 | P53 |
| Bcl-2 | Peptide/Protein bound | 5AGX | 2.24 | Bcl-2-like protein 11 |
| Bcl-2 | Peptide/Protein bound | 5AGW | 2.70 | Bcl-2-like protein 11 |
| Bcl-2 | Peptide/Protein bound | 2XA0 | 2.70 | BAX BH3 peptide |
| Bcl-2 | Peptide/Protein bound | 5JSN | 2.10 | Bcl-2 inhibitor |
| Bcl-2 | Peptide/Protein bound | 5VAY | 1.80 | Beclin 1 BH3 domain |
| Bcl-2 | Peptide/Protein bound | 5VAX | 2.00 | Beclin 1 BH3 domain |
| Bcl-2 | Peptide/Protein bound | 5VAU | 1.75 | Beclin 1 BH3 domain |
| Bcl-2 | Peptide/Protein bound | 5FCG | 2.10 | HBx-BH3 motif |
| Bcl-2 | Peptide/Protein bound | 6GQ8 | 1.90 | PUMA BH3 peptide |
| MDMX | Peptide/Protein bound | 3JZO | 1.80 | pDI Peptide (12mer) |
| MDMX | Peptide/Protein bound | 3JZP | 1.74 | pDI6W Peptide (12mer) |
| MDMX | Peptide/Protein bound | 3JZQ | 1.80 | pDIQ Peptide (12mer) |
| MDMX | Peptide/Protein bound | 5UML | 3.00 | 12-mer peptide inhibitor M3 |
| MDMX | Peptide/Protein bound | 4N5T | 1.70 | ATSP-7041 stapled peptide |
| MDMX | Peptide/Protein bound | 4RXZ | 1.55 | 12-mer peptide inhibitor |
| MDMX | Peptide/Protein bound | 3FDO | 1.40 | High affinity peptide |
| MDMX | Peptide/Protein bound | 3EQY | 1.63 | 12-mer peptide inhibitor |
| MDMX | Peptide/Protein bound | 5VK1 | 2.69 | 12-mer peptide inhibitor PMI |
| MDMX | Peptide/Protein bound | 3FEA | 1.33 | P53 peptidomimetic |
| MDMX | Peptide/Protein bound | 3DAB | 1.90 | P53 |
| Menin | Peptide/Protein bound | 4I80 | 3.10 | Macrocyclic peptidomimetic |
| Menin | Peptide/Protein bound | 3U85 | 3.00 | MLL1 |
| Menin | Peptide/Protein bound | 6OPJ | 1.50 | Peptide inhibitor 25 |
| Menin | Peptide/Protein bound | 4GQ6 | 1.55 | MLL |
| VHL | Peptide/Protein bound | 1LM8 | 1.85 | HIF-1-apha, elongin B and elongin C. |
| VHL | Peptide/Protein bound | 1LBQ | 2.00 | HIF-1-apha, elongin B and elongin C. |
| VHL | Peptide/Protein bound | 4AJY | 1.73 | HIF-1-apha, elongin B and elongin C. |
| VHL | Peptide/Protein bound | 6BVB | 2.00 | HIF-2-alpha, elongin B and elongin C. |
| VHL | Peptide/Protein bound | 6I7Q | 1.80 | HIF-2-alpha, elongin B and elongin C. |
| IL-2 | Peptide/Protein bound | 1Z92 | 2.90 | IL-2 receptor alpha chain |
| IL-2 | Peptide/Protein bound | 2B5I | 2.30 | IL-2 receptor alpha, beta and gamma chains |
| IL-2 | Peptide/Protein bound | 2ERJ | 3.00 | IL-2 receptor alpha, beta and gamma chains |
| IL-2 | Peptide/Protein bound | 5M5E | 2.30 | IL-2 receptor subunit beta and gamma. |
| XIAP | Peptide/Protein bound | 1NW9 | 2.40 | Caspase-9 |
| XIAP | Peptide/Protein bound | 1G73 | 2.00 | smac |
| XIAP | Peptide/Protein bound | 2OPZ | 3.00 | AVPF peptide |
| XIAP | Peptide/Protein bound | 2VSL | 2.10 | Smac mimetic peptide |
| XIAP | Peptide/Protein bound | 2I3H | 1.62 | 4-mer peptide |
| XIAP | Peptide/Protein bound | 1OXQ | 2.30 | Smac peptide |
| XIAP | Peptide/Protein bound | 4J48 | 2.10 | AMRV peptide |
| XIAP | Peptide/Protein bound | 4WVS | 2.09 | Peptide inhibitor |
| XIAP | Peptide/Protein bound | 4WVU | 1.96 | Peptide inhibitor |
| XIAP | Peptide/Protein bound | 1OXN | 2.20 | Peptide inhibitor |
| XIAP | Peptide/Protein bound | 1OY7 | 2.70 | Peptide inhibitor |
| XIAP | Peptide/Protein bound | 4J46 | 1.42 | AVPI peptide |
| XIAP | Peptide/Protein bound | 4J47 | 1.35 | SVPI peptide |
| XIAP | Peptide/Protein bound | 4J44 | 1.30 | AIAV peptide |
| XIAP | Peptide/Protein bound | 4J45 | 1.48 | ATAA peptide |
| Zip-A | Peptide/Protein bound | 1F47 | 1.95 | FtsZ |

**Table S4.** Output from SiteMap for all 320 protein crystal structures belonging to 12 PPI targets.

| ***PDB*** | ***Protein*** | ***Bround state*** | ***SiteScore*** | ***size*** | ***Dscore*** | ***volume*** | ***exposure*** | ***enclosure*** | ***contact*** | ***phobic*** | ***philic*** | ***balance*** | ***don/acc*** | ***refdist*** | ***refmin*** | ***refavg*** | ***sitemin*** |
| --- | --- | --- | --- | --- | --- | --- | --- | --- | --- | --- | --- | --- | --- | --- | --- | --- | --- |
| 5UFI | DCN1 | Ligand | 1.161 | 110 | 1.224 | 247.989 | 0.418 | 0.825 | 1.075 | 2.414 | 0.649 | 3.718 | 0.66 | 5.802 | 0.266 | 2.866 | 1.453 |
| 5V83 | DCN1 | Ligand | 1.12 | 99 | 1.215 | 230.839 | 0.558 | 0.715 | 0.932 | 2.105 | 0.457 | 4.608 | 8.299 | 2.417 | 0.129 | 1.268 | 0.939 |
| 5V86 | DCN1 | Ligand | 1.127 | 97 | 1.198 | 225.008 | 0.513 | 0.776 | 1.027 | 2.42 | 0.592 | 4.088 | 2.3 | 3.551 | 0.181 | 1.232 | 1.146 |
| 5V88 | DCN1 | Ligand | 1.111 | 102 | 1.202 | 221.235 | 0.597 | 0.708 | 0.875 | 1.749 | 0.498 | 3.512 | 5.216 | 3.949 | 0.207 | 1.696 | 1.31 |
| 6BG3 | DCN1 | Ligand | 1.17 | 106 | 1.253 | 219.177 | 0.475 | 0.799 | 1.083 | 2.539 | 0.511 | 4.967 | 5.103 | 3.441 | 0.209 | 1.24 | 1.222 |
| 6BG5 | DCN1 | Ligand | 1.089 | 85 | 1.163 | 238.385 | 0.583 | 0.758 | 0.975 | 2.236 | 0.487 | 4.595 | 5.798 | 3.364 | 0.088 | 1.539 | 1.195 |
| 6P5V | DCN1 | Ligand | 1.107 | 92 | 1.185 | 237.356 | 0.528 | 0.751 | 0.909 | 2.09 | 0.513 | 4.074 | 1.285 | 3.147 | 0.138 | 1.421 | 1.288 |
| 6P5W | DCN1 | Ligand | 1.133 | 96 | 1.208 | 222.607 | 0.478 | 0.779 | 1.058 | 2.188 | 0.553 | 3.958 | 1.635 | 1.764 | 0.212 | 1.022 | 1.047 |
| 3TDU | DCN1 | Peptide/protein | 1.083 | 99 | 1.15 | 214.718 | 0.531 | 0.719 | 0.98 | 1.573 | 0.664 | 2.37 | 1.398 | 2.791 | 0.065 | 1.618 | 0.5 |
| 3TDZ | DCN1 | Peptide/protein | 1.075 | 93 | 1.141 | 225.694 | 0.567 | 0.728 | 0.97 | 1.69 | 0.621 | 2.722 | 1.387 | 2.781 | 0.093 | 1.653 | 0.91 |
| 2YXJ | Bcl-xL | Ligand | 0.923 | 46 | 0.971 | 127.939 | 0.558 | 0.727 | 0.941 | 2.683 | 0.317 | 8.468 | 10.864 | 9.037 | 0.174 | 7.594 | 1.012 |
| 6VWC | Bcl-xL | Ligand | 1.059 | 194 | 1.112 | 317.275 | 0.46 | 0.713 | 1.007 | 1.576 | 0.786 | 2.007 | 0.753 | 1.81 | 0.126 | 0.815 | 1.917 |
| 7JGV | Bcl-xL | Ligand | 1.01 | 180 | 1.079 | 311.101 | 0.557 | 0.619 | 0.828 | 1.016 | 0.713 | 1.425 | 1.213 | 1.062 | 0.089 | 0.885 | 1.77 |
| 7JGW | Bcl-xL | Ligand | 1.026 | 144 | 1.088 | 307.328 | 0.598 | 0.652 | 0.864 | 1.051 | 0.746 | 1.408 | 1.015 | 1.638 | 0.225 | 1.1 | 1.777 |
| 3ZK6 | Bcl-xL | Ligand | 1.065 | 142 | 1.139 | 305.613 | 0.552 | 0.682 | 0.939 | 1.598 | 0.646 | 2.473 | 1.2 | 6.472 | 0.246 | 3.673 | 1.788 |
| 3ZLN | Bcl-xL | Ligand | 1.094 | 180 | 1.169 | 336.483 | 0.444 | 0.717 | 1.003 | 1.884 | 0.618 | 3.049 | 0.948 | 0.915 | 0.184 | 0.62 | 1.075 |
| 3ZLO | Bcl-xL | Ligand | 1.073 | 173 | 1.138 | 345.744 | 0.446 | 0.708 | 1.003 | 1.666 | 0.696 | 2.395 | 0.857 | 2.464 | 0.167 | 0.674 | 1.956 |
| 3ZLR | Bcl-xL | Ligand | 1.04 | 183 | 1.115 | 346.43 | 0.53 | 0.647 | 0.861 | 1.283 | 0.656 | 1.957 | 0.904 | 2.576 | 0.142 | 0.81 | 2.442 |
| 4C5D | Bcl-xL | Ligand | 1.036 | 51 | 1.103 | 88.151 | 0.49 | 0.817 | 1.136 | 4.656 | 0.179 | 25.942 | 2.727 | 6.07 | 0.185 | 3.775 | 0.453 |
| 4C52 | Bcl-xL | Ligand | 0.997 | 63 | 1.083 | 107.359 | 0.638 | 0.686 | 0.834 | 3.009 | 0.229 | 13.115 | 6.591 | 4.925 | 0.297 | 3.747 | 0.651 |
| 3QKD | Bcl-xL | Ligand | 0.878 | 47 | 0.905 | 158.123 | 0.621 | 0.703 | 0.881 | 2.046 | 0.496 | 4.127 | 1.338 | 8.799 | 0.151 | 8.136 | 0.593 |
| 4TUH | Bcl-xL | Ligand | 1.024 | 138 | 1.091 | 257.936 | 0.586 | 0.642 | 0.866 | 1.328 | 0.72 | 1.846 | 0.859 | 3.242 | 0.15 | 1.122 | 1.452 |
| 4QVX | Bcl-xL | Ligand | 1.052 | 175 | 1.103 | 266.854 | 0.443 | 0.707 | 1.022 | 1.581 | 0.802 | 1.973 | 1.025 | 4.258 | 0.232 | 1.203 | 1.594 |
| 4EHR | Bcl-xL | Ligand | 0.837 | 33 | 0.895 | 90.209 | 0.673 | 0.655 | 0.767 | 2.433 | 0.117 | 20.787 | 2.082 | 4.649 | 0.31 | 3.86 | 0.383 |
| 3SPF | Bcl-xL | Ligand | 0.962 | 46 | 1.036 | 154.007 | 0.596 | 0.731 | 0.89 | 2.972 | 0.123 | 24.155 | 1.524 | 6.563 | 0.151 | 4.073 | 1.088 |
| 37P7 | Bcl-xL | Ligand | 0.88 | 44 | 0.903 | 135.142 | 0.6 | 0.727 | 0.874 | 1.906 | 0.481 | 3.963 | 3.449 | 9.487 | 0.2 | 8.813 | 0.756 |
| 3CVA | Bcl-xL | Apo | 0.525 | 17 | 0.384 | 56.595 | 0.795 | 0.631 | 0.761 | 0.18 | 1.173 | 0.154 | 0.083 | 5.323 | 1.361 | 5.011 | 2.204 |
| 1R2D | Bcl-xL | Apo | 0.91 | 21 | 0.907 | 43.561 | 0.571 | 0.933 | 1.457 | 4.615 | 0.257 | 17.933 | 9.712 | 5.854 | 0.351 | 5.227 | 0.965 |
| 1R2E | Bcl-xL | Apo | 0.578 | 9 | 0.546 | 58.653 | 0.847 | 0.641 | 0.809 | 1.694 | 0.371 | 4.57 | 0.509 | 5.581 | 1.207 | 6.68 | 1.207 |
| 1R2I | Bcl-xL | Apo | 0.988 | 30 | 1.007 | 53.851 | 0.4 | 0.94 | 1.502 | 5.924 | 0.221 | 26.767 | 7.91 | 7.851 | 0.308 | 5.212 | 0.713 |
| 2P1L | Bcl-xL | Peptide/protein | 0.791 | 35 | 0.77 | 133.77 | 0.643 | 0.733 | 0.951 | 1.576 | 0.692 | 2.277 | 5.739 | 3.627 | 0.196 | 4.167 | 2.144 |
| 1PQ1 | Bcl-xL | Peptide/protein | 0.847 | 29 | 0.881 | 105.301 | 0.695 | 0.736 | 0.945 | 2.744 | 0.205 | 13.375 | 36.071 | 4.678 | 0.363 | 4.687 | 1.373 |
| 2BZW | Bcl-xL | Peptide/protein | 0.829 | 30 | 0.851 | 112.161 | 0.706 | 0.725 | 0.897 | 2.4 | 0.302 | 7.941 | 3.27 | 4.438 | 0.651 | 4.038 | 2.128 |
| 3FDL | Bcl-xL | Peptide/protein | 0.933 | 42 | 1 | 92.953 | 0.615 | 0.722 | 0.966 | 3.271 | 0.131 | 24.887 | 2.259 | 3.563 | 0.209 | 2.899 | 1.791 |
| 3JZK | HDM2 | Ligand | 0.844 | 46 | 0.869 | 196.882 | 0.731 | 0.669 | 0.849 | 1.616 | 0.521 | 3.1 | 1.318 | 1.96 | 0.133 | 2.136 | 1.013 |
| 3W69 | HDM2 | Ligand | 0.869 | 55 | 0.955 | 149.205 | 0.757 | 0.55 | 0.597 | 1.442 | 0.223 | 6.479 | 3.017 | 3.705 | 0.079 | 2.658 | 1.632 |
| 3VZV | HDM2 | Ligand | 0.814 | 33 | 0.868 | 108.731 | 0.705 | 0.634 | 0.731 | 2.467 | 0.161 | 15.323 | 3.427 | 5.516 | 0.218 | 4.598 | 1.283 |
| 4HBM | HDM2 | Ligand | 1.095 | 81 | 1.192 | 216.433 | 0.612 | 0.734 | 0.907 | 2.754 | 0.289 | 9.52 | 1.447 | 1.838 | 0.208 | 1.707 | 1.024 |
| 4JV9 | HDM2 | Ligand | 0.805 | 38 | 0.839 | 123.137 | 0.745 | 0.636 | 0.783 | 1.756 | 0.376 | 4.664 | 4.67 | 4.712 | 0.382 | 3.746 | 0.175 |
| 4JV7 | HDM2 | Ligand | 0.845 | 46 | 0.902 | 120.736 | 0.718 | 0.607 | 0.759 | 1.78 | 0.306 | 5.825 | 7.213 | 3.972 | 0.276 | 3.328 | 1.1 |
| 4JVR | HDM2 | Ligand | 0.969 | 53 | 1.051 | 173.901 | 0.681 | 0.695 | 0.802 | 2.901 | 0.154 | 18.826 | 2.492 | 3.526 | 0.288 | 2.495 | 1.008 |
| 4JVE | HDM2 | Ligand | 1.173 | 102 | 1.292 | 233.24 | 0.514 | 0.737 | 0.939 | 3.083 | 0.278 | 11.095 | 0.892 | 3.165 | 0.214 | 2.317 | 1.342 |
| 4JWR | HDM2 | Ligand | 0.93 | 40 | 0.996 | 126.91 | 0.619 | 0.728 | 0.907 | 3.239 | 0.108 | 29.88 | 1.176 | 3.34 | 0.13 | 2.398 | 0.89 |
| 4DIJ | HDM2 | Ligand | 0.893 | 52 | 0.946 | 171.5 | 0.708 | 0.652 | 0.783 | 1.854 | 0.378 | 4.905 | 2.069 | 4.506 | 0.315 | 3.494 | 2.258 |
| 4HG7 | HDM2 | Ligand | 0.911 | 47 | 0.976 | 140.287 | 0.687 | 0.675 | 0.792 | 2.372 | 0.227 | 10.435 | 2.113 | 4.688 | 0.151 | 3.181 | 1.723 |
| 4MDN | HDM2 | Ligand | 1.109 | 81 | 1.208 | 183.848 | 0.552 | 0.749 | 0.921 | 3.024 | 0.267 | 11.335 | 1.015 | 4.595 | 0.278 | 2.113 | 1.187 |
| 4MDQ | HDM2 | Ligand | 0.937 | 65.5 | 1.305 | 152.635 | 0.698 | 0.5875 | 0.7035 | 1.9245 | 0.252 | 7.6345 | 1.5515 | 6.1255 | 0.304 | 3.022 | 2.73 |
| 4ERE | HDM2 | Ligand | 0.931 | 53 | 0.981 | 160.181 | 0.632 | 0.706 | 0.861 | 2.355 | 0.389 | 6.049 | 0.733 | 3.412 | 0.219 | 2.471 | 1.354 |
| 4ERF | HDM2 | Ligand | 0.968 | 60 | 1.031 | 180.418 | 0.639 | 0.703 | 0.842 | 2.628 | 0.364 | 7.217 | 1.02 | 3.448 | 0.351 | 2.447 | 1.588 |
| 4OBA | HDM2 | Ligand | 0.994 | 56 | 1.089 | 139.944 | 0.608 | 0.694 | 0.802 | 3.132 | 0.096 | 32.579 | 0.492 | 3.56 | 0.241 | 2.361 | 1.234 |
| 4OAS | HDM2 | Ligand | 0.999 | 53 | 1.086 | 149.891 | 0.602 | 0.725 | 0.849 | 3.316 | 0.102 | 32.581 | 1.231 | 4.219 | 0.384 | 2.943 | 0.764 |
| 4OCC | HDM2 | Ligand | 0.995 | 60 | 1.073 | 174.587 | 0.634 | 0.709 | 0.834 | 2.626 | 0.246 | 10.663 | 0.561 | 3.276 | 0.157 | 2.61 | 1.177 |
| 4ODE | HDM2 | Ligand | 1.205 | 105 | 1.309 | 287.434 | 0.472 | 0.805 | 0.985 | 3.266 | 0.348 | 9.387 | 0.584 | 3.272 | 0.242 | 2.557 | 0.759 |
| 4ODF | HDM2 | Ligand | 1.136 | 86 | 1.246 | 202.027 | 0.511 | 0.746 | 0.931 | 3.127 | 0.224 | 13.957 | 2.003 | 3.287 | 0.234 | 2.303 | 0.919 |
| 4OGT | HDM2 | Ligand | 1.111 | 82 | 1.218 | 222.607 | 0.586 | 0.733 | 0.889 | 3.13 | 0.224 | 14 | 0.468 | 3.477 | 0.082 | 2.746 | 0.914 |
| 4OGV | HDM2 | Ligand | 0.996 | 55 | 1.079 | 172.529 | 0.633 | 0.721 | 0.873 | 2.996 | 0.157 | 19.053 | 1.708 | 4.232 | 0.173 | 2.681 | 0.894 |
| 4OGN | HDM2 | Ligand | 1.119 | 77 | 1.224 | 204.085 | 0.525 | 0.761 | 0.948 | 3.389 | 0.175 | 19.314 | 1.891 | 4.128 | 0.321 | 2.815 | 1.725 |
| 4OQ3 | HDM2 | Ligand | 0.916 | 54 | 0.966 | 167.384 | 0.667 | 0.682 | 0.835 | 2.179 | 0.411 | 5.3 | 0.543 | 3.371 | 0.154 | 1.816 | 1.962 |
| 4QOC | HDM2 | Ligand | 0.959 | 51 | 1.039 | 163.268 | 0.646 | 0.693 | 0.79 | 2.754 | 0.147 | 18.712 | 2.237 | 4.031 | 0.18 | 2.817 | 0.864 |
| 1RV1 | HDM2 | Ligand | 0.887 | 51 | 0.963 | 130.683 | 0.718 | 0.608 | 0.72 | 2.192 | 0.225 | 9.752 | 2.339 | 4.159 | 0.172 | 2.965 | 0.739 |
| 1T4E | HDM2 | Ligand | 0.99 | 69 | 1.062 | 202.713 | 0.657 | 0.681 | 0.809 | 1.817 | 0.391 | 4.645 | 3.862 | 1.824 | 0.387 | 1.566 | 0.872 |
| 4QO4 | HDM2 | Ligand | 0.994 | 58 | 1.07 | 184.534 | 0.623 | 0.721 | 0.851 | 2.632 | 0.239 | 11.028 | 0.963 | 3.315 | 0.105 | 2.681 | 1.159 |
| 7BIV | HDM2 | Ligand | 0.933 | 61 | 1.001 | 220.206 | 0.731 | 0.645 | 0.771 | 1.659 | 0.368 | 4.506 | 1.191 | 3.225 | 0.351 | 2.396 | 1.878 |
| 7BIT | HDM2 | Ligand | 0.993 | 70 | 1.067 | 237.356 | 0.655 | 0.68 | 0.836 | 1.959 | 0.392 | 5.003 | 2.294 | 2.172 | 0.182 | 1.961 | 0.589 |
| 7BJ6 | HDM2 | Ligand | 0.914 | 56 | 0.972 | 213.689 | 0.743 | 0.654 | 0.817 | 1.681 | 0.379 | 4.434 | 1.77 | 3.177 | 0.27 | 2.285 | 1.659 |
| 7BIR | HDM2 | Ligand | 1.038 | 85 | 1.125 | 234.269 | 0.669 | 0.664 | 0.794 | 1.738 | 0.431 | 4.035 | 1.774 | 3.464 | 0.128 | 2.089 | 1.574 |
| 7BMG | HDM2 | Ligand | 1.081 | 84 | 1.187 | 254.849 | 0.654 | 0.688 | 0.83 | 2.328 | 0.267 | 8.717 | 3.155 | 3.142 | 0.102 | 1.987 | 1.471 |
| 6Q96 | HDM2 | Ligand | 0.892 | 34 | 0.949 | 95.011 | 0.63 | 0.723 | 0.892 | 3.682 | 0.1 | 36.922 | 6.075 | 4.568 | 0.278 | 3.216 | 0.652 |
| 6Q9O | HDM2 | Ligand | 0.951 | 65 | 1.043 | 190.365 | 0.728 | 0.607 | 0.698 | 1.792 | 0.24 | 7.451 | 2.625 | 2.801 | 0.128 | 2.508 | 1.095 |
| 6Q9H | HDM2 | Ligand | 0.973 | 53 | 1.053 | 167.727 | 0.637 | 0.703 | 0.816 | 2.875 | 0.162 | 17.695 | 0.589 | 2.331 | 0.319 | 1.513 | 1.326 |
| 6Q9L | HDM2 | Ligand | 0.983 | 71 | 1.057 | 211.288 | 0.66 | 0.664 | 0.823 | 1.786 | 0.411 | 4.35 | 1.567 | 5.381 | 0.204 | 2.962 | 1.445 |
| 3LBL | HDM2 | Ligand | 1.04 | 77 | 1.153 | 211.974 | 0.696 | 0.644 | 0.751 | 2.476 | 0.179 | 13.803 | 1.306 | 2.418 | 0.3 | 1.645 | 1.02 |
| 3LBK | HDM2 | Ligand | 0.823 | 43 | 0.866 | 142.688 | 0.726 | 0.617 | 0.738 | 1.858 | 0.368 | 5.043 | 0.714 | 2.204 | 0.303 | 2.082 | 1.14 |
| 4WT2 | HDM2 | Ligand | 1.22 | 105 | 1.329 | 274.743 | 0.439 | 0.817 | 1.028 | 3.446 | 0.311 | 11.077 | 0.6 | 3.895 | 0.311 | 2.402 | 1.111 |
| 5C5A | HDM2 | Ligand | 0.89 | 49 | 0.934 | 149.891 | 0.682 | 0.679 | 0.819 | 1.905 | 0.404 | 4.718 | 3.18 | 3.895 | 0.223 | 2.86 | 0.697 |
| 5Z02 | HDM2 | Ligand | 0.948 | 47 | 1.017 | 127.596 | 0.598 | 0.715 | 0.896 | 3.077 | 0.174 | 17.687 | 5.021 | 5.275 | 0.307 | 3.271 | 1.268 |
| 5TRF | HDM2 | Ligand | 1.12 | 85 | 1.233 | 200.998 | 0.55 | 0.723 | 0.878 | 2.745 | 0.207 | 13.25 | 3.734 | 3.121 | 0.307 | 2.483 | 0.765 |
| 6GGN | HDM2 | Ligand | 0.929 | 57 | 0.99 | 167.384 | 0.702 | 0.667 | 0.806 | 2.046 | 0.367 | 5.578 | 1.015 | 2.573 | 0.106 | 1.647 | 1.3 |
| 5OAI | HDM2 | Ligand | 0.882 | 39 | 0.939 | 151.263 | 0.719 | 0.689 | 0.798 | 2.467 | 0.19 | 13.01 | 13.629 | 2.827 | 0.161 | 2.047 | 1.022 |
| 5OC8 | HDM2 | Ligand | 0.97 | 71 | 1.035 | 184.877 | 0.667 | 0.664 | 0.814 | 1.773 | 0.475 | 3.733 | 0.742 | 2.898 | 0.298 | 1.468 | 0.876 |
| 5LN2 | HDM2 | Ligand | 0.943 | 47 | 1.002 | 151.606 | 0.618 | 0.728 | 0.926 | 2.859 | 0.243 | 11.788 | 2.444 | 4.509 | 0.177 | 3.115 | 0.62 |
| 5J7G | HDM2 | Ligand | 0.971 | 63 | 1.054 | 190.365 | 0.697 | 0.659 | 0.794 | 2.206 | 0.268 | 8.244 | 3.909 | 4.569 | 0.047 | 4.114 | 0.761 |
| 5J7F | HDM2 | Ligand | 0.986 | 63 | 1.066 | 187.964 | 0.668 | 0.686 | 0.847 | 2.283 | 0.282 | 8.087 | 5.829 | 4.592 | 0.162 | 3.712 | 1.431 |
| 5LAZ | HDM2 | Ligand | 0.969 | 54 | 1.033 | 170.471 | 0.633 | 0.724 | 0.93 | 2.759 | 0.284 | 9.7 | 7.357 | 3.64 | 0.093 | 1.851 | 1.033 |
| 5LAW | HDM2 | Ligand | 1.031 | 55 | 1.119 | 147.833 | 0.534 | 0.757 | 0.953 | 3.612 | 0.099 | 36.405 | 31.692 | 3.288 | 0.308 | 2.22 | 1.336 |
| 5LAV | HDM2 | Ligand | 0.927 | 53 | 0.993 | 169.099 | 0.658 | 0.672 | 0.773 | 2.222 | 0.289 | 7.689 | 8.429 | 2.533 | 0.061 | 1.718 | 1.139 |
| 5LAY | HDM2 | Ligand | 0.918 | 63 | 0.985 | 221.235 | 0.774 | 0.619 | 0.693 | 1.351 | 0.409 | 3.305 | 2.014 | 2.413 | 0.284 | 1.874 | 0.111 |
| 5HMK | HDM2 | Ligand | 0.93 | 61 | 0.997 | 175.616 | 0.657 | 0.642 | 0.821 | 1.999 | 0.376 | 5.31 | 4.008 | 3.844 | 0.187 | 2.794 | 1.307 |
| 5HMI | HDM2 | Ligand | 0.904 | 53 | 0.974 | 144.746 | 0.717 | 0.631 | 0.719 | 2.062 | 0.271 | 7.603 | 4.24 | 3.887 | 0.354 | 3.111 | 0.858 |
| 5HMH | HDM2 | Ligand | 0.903 | 51 | 0.971 | 151.263 | 0.721 | 0.643 | 0.727 | 1.961 | 0.263 | 7.443 | 4.033 | 3.953 | 0.246 | 3.418 | 1.149 |
| 4ZFI | HDM2 | Ligand | 0.878 | 47 | 0.939 | 141.316 | 0.708 | 0.639 | 0.728 | 1.831 | 0.273 | 6.704 | 1.339 | 3.697 | 0.279 | 3.034 | 1.435 |
| 4ZGK | HDM2 | Ligand | 0.929 | 49 | 0.994 | 117.306 | 0.623 | 0.69 | 0.864 | 2.588 | 0.24 | 10.797 | 1.604 | 4.124 | 0.448 | 3.248 | 1.29 |
| 4ZYC | HDM2 | Ligand | 0.9 | 42 | 0.968 | 98.098 | 0.638 | 0.677 | 0.839 | 2.988 | 0.145 | 20.587 | 1.289 | 4.28 | 0.314 | 3.123 | 0.618 |
| 4ZYF | HDM2 | Ligand | 0.989 | 75 | 1.06 | 262.052 | 0.682 | 0.664 | 0.815 | 1.965 | 0.467 | 4.206 | 1.099 | 4.569 | 0.42 | 2.834 | 0.744 |
| 4ZYI | HDM2 | Ligand | 1.024 | 70 | 1.103 | 225.008 | 0.605 | 0.709 | 0.894 | 2.338 | 0.334 | 6.992 | 1.869 | 2.719 | 0.376 | 1.85 | 1.146 |
| 5ZXF | HDM2 | Ligand | 0.907 | 53 | 0.961 | 155.722 | 0.694 | 0.667 | 0.813 | 1.839 | 0.379 | 4.847 | 4.625 | 4.388 | 0.215 | 2.669 | 0.83 |
| 6I3S | HDM2 | Ligand | 1.018 | 63 | 1.098 | 186.249 | 0.627 | 0.724 | 0.839 | 2.807 | 0.254 | 11.04 | 2.201 | 2.472 | 0.245 | 1.445 | 0.89 |
| 3VBG | HDM2 | Ligand | 0.871 | 44 | 0.913 | 129.311 | 0.694 | 0.681 | 0.857 | 2.35 | 0.366 | 6.426 | 16.152 | 10.728 | 0.38 | 4.819 | 2.85 |
| 3VB3 | HDM2 | Apo | 0.868 | 42 | 0.927 | 136.514 | 0.704 | 0.653 | 0.808 | 2.402 | 0.225 | 10.662 | 3.275 | 5.433 | 0.165 | 3.426 | 1.021 |
| 5AFG | HDM2 | Peptide/protein | 0.911 | 59 | 0.985 | 137.2 | 0.665 | 0.612 | 0.701 | 1.879 | 0.319 | 5.888 | 2.933 | 4.347 | 0.373 | 3.18 | 0.631 |
| 4UMN | HDM2 | Peptide/protein | 0.894 | 52 | 0.948 | 168.756 | 0.677 | 0.655 | 0.778 | 2.003 | 0.376 | 5.334 | 0.775 | 5.238 | 0.245 | 3.28 | 1.396 |
| 3TPX | HDM2 | Peptide/protein | 0.952 | 66 | 1.038 | 166.355 | 0.709 | 0.618 | 0.684 | 1.889 | 0.295 | 6.398 | 7.476 | 4.494 | 0.404 | 3.088 | 0.619 |
| 3JZR | HDM2 | Peptide/protein | 0.944 | 63 | 1.009 | 216.776 | 0.721 | 0.658 | 0.785 | 1.755 | 0.404 | 4.341 | 1.191 | 5.401 | 0.243 | 3.422 | 2.272 |
| 6H22 | HDM2 | Peptide/protein | 0.895 | 54 | 0.935 | 191.737 | 0.656 | 0.672 | 0.815 | 1.807 | 0.488 | 3.701 | 1.507 | 4.528 | 0.369 | 3.555 | 1.342 |
| 6T2E | HDM2 | Peptide/protein | 0.883 | 46 | 0.949 | 136.514 | 0.671 | 0.644 | 0.778 | 2.405 | 0.23 | 10.462 | 1.239 | 5.197 | 0.375 | 3.304 | 0.769 |
| 6T2D | HDM2 | Peptide/protein | 0.79 | 20 | 0.815 | 94.668 | 0.667 | 0.729 | 0.887 | 3.306 | 0.133 | 24.93 | 1.586 | 6.359 | 0.19 | 4.604 | 0.698 |
| 5XXK | HDM2 | Peptide/protein | 0.916 | 40 | 0.968 | 137.543 | 0.626 | 0.737 | 0.879 | 2.996 | 0.212 | 14.155 | 6.877 | 6.289 | 0.383 | 3.924 | 1.572 |
| 6AAW | HDM2 | Peptide/protein | 0.914 | 57 | 0.982 | 167.727 | 0.674 | 0.635 | 0.785 | 2.054 | 0.333 | 6.165 | 9.549 | 5.249 | 0.17 | 3.211 | 1.187 |
| 6KZU | HDM2 | Peptide/protein | 0.886 | 51 | 0.948 | 149.891 | 0.698 | 0.633 | 0.729 | 2.013 | 0.317 | 6.341 | 2.167 | 5.387 | 0.31 | 3.328 | 0.635 |
| 7AD0 | HDM2 | Peptide/protein | 0.911 | 53 | 0.984 | 170.128 | 0.669 | 0.634 | 0.753 | 2.287 | 0.247 | 9.269 | 12.639 | 4.481 | 0.205 | 3.198 | 0.351 |
| 5VK0 | HDM2 | Peptide/protein | 0.916 | 53 | 0.978 | 185.563 | 0.694 | 0.662 | 0.804 | 2.116 | 0.317 | 6.667 | 4.101 | 5.467 | 0.014 | 3.471 | 1.377 |
| 5UMM | HDM2 | Peptide/protein | 0.849 | 48 | 0.897 | 159.152 | 0.694 | 0.621 | 0.775 | 1.899 | 0.391 | 4.857 | 3.816 | 3.995 | 0.209 | 3.047 | 0.745 |
| 2GV2 | HDM2 | Peptide/protein | 0.865 | 44 | 0.919 | 164.297 | 0.718 | 0.649 | 0.779 | 2.085 | 0.288 | 7.244 | 15.203 | 5.28 | 0.136 | 3.49 | 1.296 |
| 6Y4Q | HDM2 | Peptide/protein | 0.978 | 77 | 1.076 | 199.626 | 0.708 | 0.593 | 0.688 | 1.636 | 0.322 | 5.084 | 3.948 | 4.556 | 0.23 | 2.833 | 1.79 |
| 2AXI | HDM2 | Peptide/protein | 0.936 | 57 | 1.027 | 155.379 | 0.721 | 0.621 | 0.685 | 2.157 | 0.169 | 12.745 | 6.905 | 5.178 | 0.268 | 3.262 | 0.679 |
| 4UE1 | HDM2 | Peptide/protein | 0.905 | 51 | 0.974 | 157.437 | 0.675 | 0.644 | 0.775 | 2.203 | 0.257 | 8.586 | 2.409 | 3.986 | 0.203 | 3.117 | 0.726 |
| 4UD7 | HDM2 | Peptide/protein | 0.922 | 53 | 0.994 | 160.181 | 0.686 | 0.652 | 0.789 | 2.328 | 0.25 | 9.301 | 3.863 | 3.977 | 0.211 | 3.012 | 0.72 |
| 4HFZ | HDM2 | Peptide/protein | 0.847 | 38 | 0.901 | 137.2 | 0.701 | 0.652 | 0.78 | 2.184 | 0.214 | 10.212 | 46.109 | 4.029 | 0.364 | 3.44 | 1.482 |
| 6HFA | HDM2 | Peptide/protein | 0.852 | 51 | 0.89 | 153.664 | 0.669 | 0.634 | 0.799 | 1.835 | 0.496 | 3.697 | 1.003 | 4.24 | 0.272 | 3.35 | 0.638 |
| 6IM9 | HDM2 | Peptide/protein | 0.938 | 58 | 1.018 | 167.041 | 0.681 | 0.638 | 0.73 | 2.092 | 0.249 | 8.416 | 10.388 | 6.019 | 0.298 | 3.668 | 2.18 |
| 1YCR | HDM2 | Peptide/protein | 0.89 | 57 | 0.964 | 151.263 | 0.674 | 0.592 | 0.727 | 2.066 | 0.309 | 6.677 | 6.272 | 4.833 | 0.319 | 3.259 | 0.642 |
| 4IPF | XDM2 | Ligand | 0.848 | 44 | 0.895 | 132.741 | 0.712 | 0.64 | 0.761 | 1.839 | 0.345 | 5.337 | 9.166 | 5.511 | 0.378 | 4.317 | 0.823 |
| 4J3E | XDM2 | Ligand | 0.873 | 54 | 0.927 | 143.374 | 0.73 | 0.618 | 0.739 | 1.555 | 0.413 | 3.766 | 6.734 | 3.386 | 0.374 | 2.684 | 0.752 |
| 4J74 | XDM2 | Ligand | 0.82 | 37 | 0.853 | 108.731 | 0.684 | 0.663 | 0.757 | 2.049 | 0.359 | 5.705 | 8.694 | 2.811 | 0.11 | 2.048 | 0.893 |
| 4JRG | XDM2 | Ligand | 0.902 | 49 | 0.978 | 107.016 | 0.682 | 0.633 | 0.739 | 2.202 | 0.182 | 12.111 | 1.717 | 4.313 | 0.258 | 3.24 | 1.144 |
| 4JSC | XDM2 | Ligand | 0.94 | 56 | 1.03 | 132.055 | 0.687 | 0.631 | 0.702 | 2.356 | 0.159 | 14.843 | 0.971 | 4.19 | 0.168 | 2.971 | 1.087 |
| 4J7D | XDM2 | Ligand | 0.883 | 53 | 0.935 | 144.06 | 0.699 | 0.64 | 0.756 | 1.776 | 0.408 | 4.357 | 10.261 | 3.534 | 0.51 | 2.545 | 0.808 |
| 4J7E | XDM2 | Ligand | 0.801 | 36 | 0.834 | 102.9 | 0.684 | 0.643 | 0.701 | 1.738 | 0.356 | 4.887 | 5.868 | 5.75 | 0.335 | 5.181 | 1.665 |
| 4LWT | XDM2 | Ligand | 0.863 | 41 | 0.901 | 105.644 | 0.631 | 0.69 | 0.793 | 2.526 | 0.353 | 7.153 | 4.138 | 3.781 | 0.306 | 3.303 | 1.148 |
| 4LWU | XDM2 | Ligand | 0.944 | 52 | 1.03 | 121.422 | 0.683 | 0.661 | 0.767 | 2.645 | 0.136 | 19.419 | 1.836 | 4.427 | 0.271 | 3.24 | 1.291 |
| 4LWV | XDM2 | Ligand | 0.991 | 57 | 1.084 | 146.118 | 0.61 | 0.689 | 0.856 | 2.934 | 0.12 | 24.541 | 4.026 | 4.173 | 0.341 | 3.27 | 1.585 |
| 1YCQ | XDM2 | Peptide/protein | 0.824 | 27 | 0.85 | 83.692 | 0.565 | 0.728 | 0.981 | 3.278 | 0.23 | 14.274 | 9.711 | 5.107 | 0.269 | 3.72 | 0.467 |
| 6QGH | Bcl-2 | Ligand | 0.907 | 60 | 0.986 | 132.398 | 0.689 | 0.593 | 0.627 | 1.899 | 0.302 | 6.299 | 2.132 | 9.64 | 0.185 | 7.55 | 1.007 |
| 6QGK | Bcl-2 | Ligand | 0.906 | 57 | 0.969 | 196.196 | 0.752 | 0.634 | 0.731 | 1.462 | 0.372 | 3.932 | 3.155 | 1.827 | 0.253 | 2.151 | 1.393 |
| 6QGJ | Bcl-2 | Ligand | 0.98 | 61 | 1.066 | 188.993 | 0.686 | 0.673 | 0.808 | 2.326 | 0.219 | 10.605 | 2.877 | 10.57 | 0.434 | 9.337 | 1.511 |
| 7LHB | Bcl-2 | Ligand | 0.938 | 50 | 0.996 | 126.224 | 0.597 | 0.713 | 0.872 | 2.694 | 0.297 | 9.076 | 2.533 | 8.116 | 0.413 | 8.207 | 1.172 |
| 4IEH | Bcl-2 | Ligand | 0.861 | 45 | 0.891 | 125.881 | 0.656 | 0.685 | 0.786 | 1.892 | 0.46 | 4.111 | 2.738 | 8.84 | 0.053 | 6.73 | 0.81 |
| 2W3L | Bcl-2 | Ligand | 0.745 | 20 | 0.772 | 89.866 | 0.753 | 0.667 | 0.752 | 2.138 | 0.15 | 14.216 | 3.593 | 3.788 | 0.068 | 3.583 | 0.949 |
| 4LVT | Bcl-2 | Ligand | 0.812 | 43 | 0.835 | 139.258 | 0.699 | 0.643 | 0.835 | 2.017 | 0.514 | 3.926 | 1.846 | 10.013 | 0.35 | 9.157 | 1.16 |
| 4LXD | Bcl-2 | Ligand | 0.94 | 57 | 0.997 | 157.094 | 0.558 | 0.69 | 0.849 | 2.226 | 0.389 | 5.724 | 2.905 | 8.118 | 0.122 | 7.548 | 1.343 |
| 4MAN | Bcl-2 | Ligand | 0.902 | 52 | 0.942 | 146.118 | 0.626 | 0.69 | 0.832 | 2.038 | 0.461 | 4.418 | 1.953 | 7.768 | 0.26 | 6.47 | 0.716 |
| 6GL8 | Bcl-2 | Ligand | 0.856 | 46 | 0.925 | 164.297 | 0.788 | 0.601 | 0.616 | 1.581 | 0.223 | 7.099 | 2.009 | 4.064 | 0.298 | 3.608 | 0.524 |
| 6O0P | Bcl-2 | Ligand | 0.955 | 52 | 1.001 | 121.765 | 0.532 | 0.748 | 0.936 | 2.882 | 0.384 | 7.509 | 3.567 | 7.724 | 0.145 | 5.9 | 1.161 |
| 6O0O | Bcl-2 | Ligand | 0.845 | 38 | 0.907 | 120.736 | 0.775 | 0.634 | 0.626 | 1.946 | 0.161 | 12.065 | 3.344 | 3.693 | 0.19 | 3.878 | 0.961 |
| 6O0L | Bcl-2 | Ligand | 0.98 | 54 | 1.043 | 122.108 | 0.546 | 0.743 | 0.921 | 3.226 | 0.287 | 11.234 | 1.656 | 7.79 | 0.219 | 6.144 | 0.638 |
| 6O0K | Bcl-2 | Ligand | 0.963 | 46 | 1.012 | 116.62 | 0.505 | 0.778 | 1.007 | 3.486 | 0.285 | 12.251 | 2.924 | 7.891 | 0.203 | 6.312 | 0.973 |
| 6O0M | Bcl-2 | Ligand | 1.096 | 104 | 1.194 | 362.551 | 0.61 | 0.674 | 0.839 | 1.749 | 0.461 | 3.795 | 1.027 | 2.003 | 0.268 | 1.762 | 2.952 |
| 4AQ3 | Bcl-2 | Ligand | 0.857 | 38 | 0.909 | 109.417 | 0.683 | 0.669 | 0.779 | 2.248 | 0.221 | 10.15 | 1.923 | 3.77 | 0.148 | 3.792 | 0.726 |
| 6QGG | Bcl-2 | Ligand | 0.889 | 41 | 0.926 | 114.219 | 0.602 | 0.726 | 0.887 | 2.675 | 0.342 | 7.817 | 6.998 | 9.03 | 0.201 | 7.092 | 1.091 |
| 5AGX | Bcl-2 | Peptide/protein | 0.908 | 55 | 0.944 | 229.467 | 0.707 | 0.696 | 0.885 | 1.625 | 0.525 | 3.098 | 2.524 | 5.108 | 0.212 | 3.86 | 1.091 |
| 5AGW | Bcl-2 | Peptide/protein | 0.778 | 28 | 0.823 | 84.035 | 0.767 | 0.63 | 0.689 | 1.879 | 0.161 | 11.649 | 1.987 | 3.345 | 0.443 | 3.399 | 0.987 |
| 2XA0 | Bcl-2 | Peptide/protein | 0.801 | 48 | 0.839 | 219.52 | 0.794 | 0.58 | 0.721 | 1.089 | 0.49 | 2.222 | 0.978 | 5.127 | 0.302 | 3.174 | 2.117 |
| 5JSN | Bcl-2 | Peptide/protein | 0.812 | 32 | 0.858 | 101.528 | 0.74 | 0.65 | 0.74 | 2.127 | 0.194 | 10.983 | 7.382 | 2.98 | 0.224 | 3.22 | 1.105 |
| 5VAY | Bcl-2 | Peptide/protein | 0.829 | 45 | 0.869 | 137.543 | 0.704 | 0.626 | 0.774 | 1.644 | 0.421 | 3.902 | 0.588 | 5.432 | 0.413 | 3.534 | 2.463 |
| 5VAX | Bcl-2 | Peptide/protein | 0.74 | 20 | 0.767 | 85.75 | 0.767 | 0.658 | 0.725 | 2.187 | 0.147 | 14.89 | 1.232 | 3.797 | 0.297 | 3.842 | 0.321 |
| 5VAU | Bcl-2 | Peptide/protein | 0.8 | 28 | 0.806 | 128.282 | 0.654 | 0.73 | 0.924 | 2.084 | 0.397 | 5.248 | 0.349 | 4.081 | 0.419 | 3.971 | 1.101 |
| 5FCG | Bcl-2 | Peptide/protein | 0.728 | 24 | 0.739 | 138.915 | 0.782 | 0.649 | 0.772 | 1.403 | 0.339 | 4.137 | 2.147 | 5.29 | 0.485 | 4.013 | 2.489 |
| 6GQ8 | Bcl-2 | Peptide/protein | 0.814 | 37 | 0.858 | 139.944 | 0.739 | 0.631 | 0.779 | 2.019 | 0.281 | 7.182 | 1.869 | 4.274 | 0.329 | 3.22 | 1.546 |
| 7C3Y | MDMX | Ligand | 0.782 | 38 | 0.819 | 98.441 | 0.701 | 0.597 | 0.674 | 1.478 | 0.363 | 4.07 | 4.964 | 3.247 | 0.101 | 2.847 | 1.544 |
| 7C3Q | MDMX | Ligand | 0.738 | 30 | 0.755 | 99.47 | 0.746 | 0.617 | 0.75 | 1.499 | 0.399 | 3.757 | 15.83 | 3.186 | 0.229 | 3.23 | 1.48 |
| 7C44 | MDMX | Ligand | 0.758 | 35 | 0.789 | 99.127 | 0.722 | 0.591 | 0.673 | 1.416 | 0.372 | 3.805 | 4.664 | 3.061 | 0.094 | 3.056 | 1.261 |
| 6Q9W | MDMX | Ligand | 0.945 | 60 | 1.022 | 164.297 | 0.672 | 0.647 | 0.768 | 1.864 | 0.289 | 6.443 | 10.538 | 1.999 | 0.151 | 1.959 | 0.543 |
| 6Q9Y | MDMX | Ligand | 0.776 | 28 | 0.836 | 90.552 | 0.731 | 0.598 | 0.704 | 2.581 | 0.061 | 42.655 | 4.365 | 4.653 | 0.282 | 2.13 | 0.724 |
| 6Q9Q | MDMX | Ligand | 0.896 | 48 | 0.957 | 141.659 | 0.631 | 0.658 | 0.788 | 2.091 | 0.273 | 7.672 | 4.149 | 5.455 | 0.256 | 3.494 | 0.491 |
| 6Q9S | MDMX | Ligand | 1.108 | 109 | 1.19 | 428.407 | 0.56 | 0.723 | 0.886 | 1.474 | 0.563 | 2.619 | 1.07 | 3.821 | 0.273 | 1.965 | 1.302 |
| 6Q9U | MDMX | Ligand | 0.77 | 30 | 0.793 | 101.528 | 0.714 | 0.651 | 0.83 | 1.955 | 0.346 | 5.653 | 26.892 | 3.711 | 0.379 | 2.477 | 1.279 |
| 3LBJ | MDMX | Ligand | 0.82 | 41 | 0.855 | 113.533 | 0.682 | 0.638 | 0.815 | 1.981 | 0.398 | 4.977 | 3.221 | 3.698 | 0.218 | 2.975 | 1.616 |
| 3U15 | MDMX | Ligand | 0.814 | 29 | 0.86 | 80.605 | 0.67 | 0.668 | 0.807 | 2.527 | 0.145 | 17.446 | 1.745 | 11.209 | 0.452 | 6.025 | 3.315 |
| 3JZO | MDMX | Peptide/protein | 0.838 | 40 | 0.881 | 157.437 | 0.677 | 0.65 | 0.821 | 1.844 | 0.317 | 5.822 | 22.31 | 1.189 | 0.297 | 1.301 | 0.229 |
| 3JZP | MDMX | Peptide/protein | 0.759 | 30 | 0.779 | 140.973 | 0.754 | 0.639 | 0.766 | 1.49 | 0.364 | 4.09 | 18.727 | 0.9 | 0.33 | 1.467 | 0.704 |
| 3JZQ | MDMX | Peptide/protein | 0.801 | 42 | 0.838 | 158.123 | 0.738 | 0.606 | 0.739 | 1.178 | 0.415 | 2.839 | 18.164 | 0.869 | 0.274 | 1.072 | 0.299 |
| 5UML | MDMX | Peptide/protein | 0.846 | 43 | 0.897 | 166.698 | 0.757 | 0.634 | 0.749 | 1.598 | 0.308 | 5.194 | 9.522 | 1.52 | 0.109 | 1.226 | 0.678 |
| 4N5T | MDMX | Peptide/protein | 0.884 | 38 | 0.937 | 125.881 | 0.642 | 0.703 | 0.886 | 2.702 | 0.197 | 13.728 | 11.357 | 2.644 | 0.27 | 1.535 | 1.099 |
| 4RXZ | MDMX | Peptide/protein | 0.907 | 54 | 0.955 | 180.761 | 0.64 | 0.672 | 0.887 | 1.711 | 0.426 | 4.021 | 7.061 | 1.977 | 0.233 | 1.256 | 1.005 |
| 3FDO | MDMX | Peptide/protein | 0.79 | 35 | 0.816 | 156.065 | 0.727 | 0.644 | 0.798 | 1.753 | 0.388 | 4.522 | 10.674 | 1.382 | 0.221 | 1.293 | 1.008 |
| 3EQY | MDMX | Peptide/protein | 0.909 | 40 | 0.97 | 122.794 | 0.545 | 0.711 | 0.986 | 3.211 | 0.156 | 20.582 | 11.358 | 4.948 | 0.456 | 2.517 | 0.652 |
| 5VK1 | MDMX | Peptide/protein | 0.803 | 43 | 0.855 | 146.461 | 0.753 | 0.576 | 0.686 | 1.495 | 0.328 | 4.553 | 9.441 | 1.681 | 0.163 | 1.305 | 0.842 |
| 3FEA | MDMX | Peptide/protein | 0.929 | 54 | 1.014 | 152.978 | 0.597 | 0.632 | 0.84 | 2.705 | 0.171 | 15.772 | 4.526 | 4.332 | 0.14 | 1.358 | 0.957 |
| 3DAB | MDMX | Peptide/protein | 0.9 | 37 | 0.958 | 110.789 | 0.565 | 0.718 | 0.977 | 3.078 | 0.137 | 22.445 | 3.912 | 4.483 | 0.056 | 1.973 | 0.707 |
| 1R6N | HPV E2 | Ligand | 0.823 | 46 | 0.839 | 138.229 | 0.681 | 0.657 | 0.824 | 1.227 | 0.592 | 2.071 | 0.529 | 3.204 | 0.427 | 2.457 | 1.408 |
| 1R6K | HPV E2 | Apo | 0.748 | 41 | 0.7 | 120.393 | 0.62 | 0.702 | 1.047 | 0.896 | 0.992 | 0.903 | 0.574 | 3.84 | 0.144 | 3.705 | 1.01 |
| 4GQ3 | Menin | Ligand | 0.737 | 33 | 0.677 | 194.824 | 0.756 | 0.745 | 0.758 | 0.194 | 0.95 | 0.204 | 0.866 | 5.311 | 0.329 | 3.294 | 1.052 |
| 4GQ4 | Menin | Ligand | 0.736 | 34 | 0.675 | 192.08 | 0.755 | 0.743 | 0.761 | 0.234 | 0.979 | 0.239 | 0.82 | 5.42 | 0.335 | 3.447 | 0.981 |
| 4X5Y | Menin | Ligand | 0.819 | 55 | 0.786 | 322.077 | 0.802 | 0.709 | 0.714 | 0.1 | 1.033 | 0.097 | 0.785 | 8.006 | 0.263 | 6.566 | 1.55 |
| 4X5Z | Menin | Ligand | 0.802 | 49 | 0.764 | 328.251 | 0.814 | 0.717 | 0.731 | 0.161 | 0.994 | 0.162 | 0.803 | 6.658 | 0.352 | 4.968 | 1.457 |
| 4OG3 | Menin | Ligand | 0.918 | 72 | 0.916 | 342.314 | 0.755 | 0.717 | 0.777 | 0.371 | 0.958 | 0.387 | 1.047 | 5.194 | 0.168 | 4.623 | 0.862 |
| 4OG4 | Menin | Ligand | 0.794 | 34 | 0.749 | 204.085 | 0.738 | 0.788 | 0.972 | 0.719 | 0.836 | 0.859 | 1.878 | 5.506 | 0.24 | 5.148 | 1.137 |
| 4OG5 | Menin | Ligand | 0.727 | 29 | 0.679 | 222.607 | 0.825 | 0.733 | 0.718 | 0.269 | 0.82 | 0.328 | 0.983 | 4.412 | 0.349 | 4.137 | 0.958 |
| 4OG6 | Menin | Ligand | 1.01 | 100 | 1.021 | 397.194 | 0.731 | 0.714 | 0.773 | 0.2 | 1.066 | 0.187 | 0.842 | 5.802 | 0.124 | 2.963 | 0.43 |
| 4OG7 | Menin | Ligand | 0.736 | 31 | 0.696 | 227.066 | 0.815 | 0.718 | 0.685 | 0.186 | 0.792 | 0.235 | 1.088 | 4.943 | 0.149 | 4.755 | 1.182 |
| 4OG8 | Menin | Ligand | 0.885 | 52 | 0.889 | 257.936 | 0.769 | 0.737 | 0.767 | 0.737 | 0.71 | 1.037 | 1.366 | 5.018 | 0.02 | 2.97 | 1.43 |
| 6O5I | Menin | Ligand | 0.832 | 55 | 0.799 | 345.744 | 0.808 | 0.722 | 0.735 | 0.14 | 1.015 | 0.137 | 0.747 | 8.242 | 0.189 | 7.077 | 1.513 |
| 6S2K | Menin | Ligand | 0.533 | 17 | 0.429 | 94.668 | 0.832 | 0.644 | 0.755 | 0.264 | 1.058 | 0.25 | 0.939 | 3.987 | 0.453 | 3.179 | 0.975 |
| 6PKC | Menin | Ligand | 0.809 | 46 | 0.778 | 260.68 | 0.794 | 0.726 | 0.747 | 0.203 | 0.905 | 0.225 | 0.877 | 5.595 | 0.157 | 5.003 | 1.263 |
| 6EA1 | Menin | Ligand | 0.963 | 87 | 0.947 | 451.045 | 0.777 | 0.716 | 0.792 | 0.204 | 1.102 | 0.185 | 0.971 | 6.628 | 0.288 | 5.023 | 0.625 |
| 6B41 | Menin | Ligand | 0.773 | 38 | 0.729 | 268.912 | 0.808 | 0.74 | 0.777 | 0.335 | 0.904 | 0.371 | 1.423 | 8.817 | 0.121 | 7.52 | 0.654 |
| 5DB0 | Menin | Ligand | 0.787 | 40 | 0.751 | 246.96 | 0.806 | 0.733 | 0.706 | 0.155 | 0.87 | 0.178 | 1.583 | 7.549 | 0.189 | 6.084 | 1.089 |
| 5DB1 | Menin | Ligand | 0.838 | 58 | 0.809 | 328.937 | 0.794 | 0.711 | 0.735 | 0.166 | 1.024 | 0.162 | 0.843 | 6.607 | 0.209 | 5.243 | 1.483 |
| 5DB2 | Menin | Ligand | 0.762 | 42 | 0.691 | 283.661 | 0.814 | 0.728 | 0.746 | 0.082 | 1.088 | 0.075 | 1.018 | 7.535 | 0.297 | 6.507 | 1.031 |
| 5DB3 | Menin | Ligand | 0.818 | 52 | 0.792 | 327.565 | 0.821 | 0.705 | 0.677 | 0.135 | 0.948 | 0.143 | 0.93 | 7.785 | 0.282 | 4.408 | 1.435 |
| 5DDF | Menin | Ligand | 0.769 | 45 | 0.721 | 297.038 | 0.814 | 0.712 | 0.731 | 0.125 | 1.034 | 0.121 | 0.585 | 5.373 | 0.154 | 4.087 | 1.4 |
| 5DDE | Menin | Ligand | 0.846 | 61 | 0.804 | 313.159 | 0.782 | 0.708 | 0.749 | 0.091 | 1.089 | 0.083 | 0.685 | 5.798 | 0.231 | 3.958 | 1.346 |
| 5DDA | Menin | Ligand | 0.788 | 40 | 0.747 | 217.462 | 0.732 | 0.745 | 0.795 | 0.202 | 0.904 | 0.223 | 0.85 | 4.326 | 0.085 | 3.669 | 1.231 |
| 5DDB | Menin | Ligand | 0.845 | 60 | 0.8 | 298.753 | 0.768 | 0.713 | 0.79 | 0.127 | 1.092 | 0.116 | 0.7 | 5.004 | 0.166 | 3.153 | 1.427 |
| 5DDC | Menin | Ligand | 0.831 | 56 | 0.799 | 308.7 | 0.791 | 0.715 | 0.75 | 0.125 | 1.021 | 0.122 | 0.762 | 4.488 | 0.183 | 3.112 | 1.549 |
| 5DDD | Menin | Ligand | 0.73 | 31 | 0.673 | 197.225 | 0.777 | 0.74 | 0.741 | 0.206 | 0.903 | 0.228 | 0.69 | 5.326 | 0.241 | 3.387 | 1.14 |
| 5DD9 | Menin | Ligand | 0.793 | 50 | 0.732 | 273.714 | 0.772 | 0.71 | 0.762 | 0.136 | 1.099 | 0.123 | 0.527 | 4.672 | 0.179 | 3.661 | 0.962 |
| 6BXH | Menin | Ligand | 0.805 | 43 | 0.771 | 256.907 | 0.796 | 0.738 | 0.744 | 0.192 | 0.882 | 0.217 | 1.03 | 9.751 | 0.276 | 7.545 | 1.41 |
| 6BY8 | Menin | Ligand | 0.798 | 49 | 0.759 | 320.705 | 0.823 | 0.713 | 0.712 | 0.114 | 1.002 | 0.113 | 0.972 | 8.437 | 0.205 | 7.155 | 1.533 |
| 6BXY | Menin | Ligand | 0.793 | 41 | 0.763 | 233.926 | 0.808 | 0.726 | 0.696 | 0.133 | 0.842 | 0.158 | 1.459 | 8.253 | 0.198 | 6.715 | 0.948 |
| 3U84 | Menin | Apo | 0.809 | 45 | 0.785 | 235.298 | 0.777 | 0.717 | 0.784 | 0.336 | 0.846 | 0.397 | 1.508 | 8.55 | 0.26 | 6.025 | 0.995 |
| 4I80 | Menin | Protein/Peptide | 0.927 | 79 | 0.917 | 358.092 | 0.726 | 0.71 | 0.794 | 0.171 | 1.057 | 0.162 | 0.934 | 8.608 | 0.081 | 6.714 | 2.333 |
| 3U85 | Menin | Protein/Peptide | 0.913 | 76 | 0.876 | 353.976 | 0.749 | 0.709 | 0.812 | 0.097 | 1.132 | 0.086 | 1.029 | 9.417 | 0.108 | 6.727 | 3.128 |
| 6OPJ | Menin | Protein/Peptide | 0.889 | 66 | 0.879 | 339.913 | 0.782 | 0.714 | 0.766 | 0.175 | 0.96 | 0.182 | 0.929 | 8.26 | 0.163 | 6.575 | 1.135 |
| 4GQ6 | Menin | Protein/Peptide | 0.751 | 37 | 0.699 | 202.37 | 0.774 | 0.729 | 0.763 | 0.105 | 0.951 | 0.11 | 0.974 | 8.612 | 0.217 | 6.637 | 0.895 |
| 3ZRC | VHL | Ligand | 0.609 | 23 | 0.586 | 78.89 | 0.787 | 0.565 | 0.755 | 0.795 | 0.626 | 1.269 | 0.717 | 2.418 | 0.463 | 2.353 | 1.33 |
| 3ZTC | VHL | Ligand | 0.615 | 21 | 0.588 | 41.846 | 0.691 | 0.599 | 0.83 | 1.54 | 0.603 | 2.553 | 0.529 | 7.771 | 0.335 | 5.069 | 1.138 |
| 3ZTD | VHL | Ligand | 0.536 | 16 | 0.458 | 55.223 | 0.742 | 0.616 | 0.833 | 0.51 | 0.883 | 0.578 | 1.537 | 2.542 | 0.316 | 3.043 | 1.111 |
| 3ZUN | VHL | Ligand | 0.442 | 10 | 0.327 | 51.45 | 0.787 | 0.611 | 0.862 | 0.05 | 1.033 | 0.048 | 1.821 | 2.349 | 0.503 | 3.988 | 0.988 |
| 4B9K | VHL | Ligand | 0.731 | 45 | 0.718 | 86.093 | 0.667 | 0.594 | 0.837 | 0.964 | 0.825 | 1.169 | 0.945 | 5.123 | 0.154 | 2.159 | 1.181 |
| 4B95 | VHL | Ligand | 0.635 | 32 | 0.597 | 72.03 | 0.724 | 0.578 | 0.794 | 0.49 | 0.866 | 0.566 | 0.451 | 1.528 | 0.224 | 1.732 | 0.607 |
| 4BKS | VHL | Ligand | 0.64 | 30 | 0.588 | 87.465 | 0.706 | 0.621 | 0.962 | 0.962 | 0.92 | 1.046 | 1.095 | 3.509 | 0.272 | 1.887 | 0.765 |
| 4BKT | VHL | Ligand | 0.526 | 15 | 0.465 | 50.764 | 0.776 | 0.58 | 0.775 | 0.398 | 0.756 | 0.526 | 0.952 | 2.147 | 0.431 | 2.855 | 1.088 |
| 4W9C | VHL | Ligand | 0.713 | 41 | 0.7 | 85.064 | 0.637 | 0.589 | 0.82 | 0.862 | 0.784 | 1.1 | 1.131 | 4.047 | 0.175 | 2.185 | 0.954 |
| 4W9D | VHL | Ligand | 0.667 | 34 | 0.648 | 97.412 | 0.734 | 0.571 | 0.795 | 0.752 | 0.745 | 1.009 | 0.733 | 3.483 | 0.289 | 1.949 | 1.24 |
| 4W9E | VHL | Ligand | 0.703 | 38 | 0.7 | 82.663 | 0.71 | 0.569 | 0.738 | 0.915 | 0.677 | 1.351 | 1.619 | 3.514 | 0.289 | 2.095 | 1.449 |
| 4W9F | VHL | Ligand | 0.726 | 45 | 0.715 | 89.523 | 0.656 | 0.585 | 0.814 | 0.871 | 0.819 | 1.064 | 1.098 | 4.019 | 0.184 | 2.172 | 0.789 |
| 4W9G | VHL | Ligand | 0.75 | 52 | 0.737 | 100.842 | 0.641 | 0.591 | 0.851 | 0.891 | 0.906 | 0.983 | 0.606 | 2.223 | 0.311 | 2.22 | 1.173 |
| 4W9H | VHL | Ligand | 0.719 | 42 | 0.702 | 88.494 | 0.653 | 0.599 | 0.861 | 0.862 | 0.818 | 1.054 | 1.005 | 5.939 | 0.393 | 2.711 | 1.099 |
| 4W9I | VHL | Ligand | 0.699 | 39 | 0.679 | 89.523 | 0.672 | 0.593 | 0.824 | 0.825 | 0.81 | 1.018 | 0.903 | 5.111 | 0.289 | 2.293 | 1.148 |
| 4W9J | VHL | Ligand | 0.731 | 41 | 0.723 | 90.895 | 0.643 | 0.605 | 0.848 | 0.923 | 0.743 | 1.243 | 1.015 | 6.926 | 0.338 | 3.606 | 0.95 |
| 4W9K | VHL | Ligand | 0.734 | 45 | 0.715 | 87.808 | 0.622 | 0.61 | 0.877 | 0.808 | 0.863 | 0.937 | 0.904 | 7.473 | 0.325 | 3.878 | 0.9 |
| 4W9L | VHL | Ligand | 0.74 | 47 | 0.743 | 107.359 | 0.695 | 0.57 | 0.784 | 0.841 | 0.746 | 1.129 | 0.773 | 5.487 | 0.393 | 2.465 | 1.513 |
| 5LLI | VHL | Ligand | 0.724 | 45 | 0.702 | 101.871 | 0.656 | 0.602 | 0.83 | 0.913 | 0.888 | 1.029 | 0.883 | 5.855 | 0.263 | 2.797 | 0.965 |
| 5NVV | VHL | Ligand | 0.685 | 31 | 0.663 | 41.846 | 0.608 | 0.616 | 0.874 | 1.607 | 0.705 | 2.279 | 0.911 | 8.717 | 0.375 | 5.966 | 1 |
| 5NVW | VHL | Ligand | 0.664 | 35 | 0.638 | 95.697 | 0.713 | 0.576 | 0.812 | 0.816 | 0.807 | 1.01 | 0.921 | 4.928 | 0.366 | 3.026 | 0.946 |
| 5NVY | VHL | Ligand | 0.681 | 37 | 0.654 | 89.523 | 0.711 | 0.593 | 0.819 | 0.871 | 0.839 | 1.038 | 1.048 | 2.931 | 0.212 | 2.077 | 1.47 |
| 5NVZ | VHL | Ligand | 0.712 | 45 | 0.682 | 113.533 | 0.696 | 0.603 | 0.856 | 0.805 | 0.953 | 0.844 | 0.517 | 4.563 | 0.302 | 2.437 | 1.261 |
| 5NW0 | VHL | Ligand | 0.711 | 38 | 0.692 | 96.726 | 0.681 | 0.611 | 0.868 | 0.983 | 0.78 | 1.26 | 1.074 | 7.064 | 0.37 | 3.341 | 1.244 |
| 5NW1 | VHL | Ligand | 0.732 | 42 | 0.718 | 95.011 | 0.656 | 0.611 | 0.889 | 1.09 | 0.789 | 1.382 | 1.358 | 7.036 | 0.437 | 3.289 | 1.316 |
| 5NW2 | VHL | Ligand | 0.725 | 42 | 0.71 | 97.069 | 0.661 | 0.605 | 0.852 | 0.915 | 0.804 | 1.139 | 0.886 | 5.048 | 0.223 | 2.784 | 1.055 |
| 6FMI | VHL | Ligand | 0.784 | 52 | 0.778 | 95.354 | 0.591 | 0.622 | 0.941 | 0.906 | 0.836 | 1.083 | 0.785 | 3.868 | 0.384 | 2.006 | 1.096 |
| 6FMJ | VHL | Ligand | 0.701 | 39 | 0.675 | 95.011 | 0.678 | 0.607 | 0.841 | 0.858 | 0.849 | 1.011 | 0.863 | 3.921 | 0.258 | 2.195 | 1.062 |
| 6FMK | VHL | Ligand | 0.639 | 27 | 0.604 | 84.721 | 0.73 | 0.603 | 0.82 | 1.103 | 0.756 | 1.459 | 1.174 | 4.577 | 0.289 | 2.552 | 1.081 |
| 6HAY | VHL | Ligand | 0.75 | 45 | 0.727 | 97.755 | 0.583 | 0.639 | 0.934 | 0.814 | 0.88 | 0.924 | 1.396 | 6.727 | 0.317 | 5.445 | 1.348 |
| 6HR2 | VHL | Ligand | 0.724 | 40 | 0.694 | 104.958 | 0.633 | 0.639 | 0.878 | 0.92 | 0.872 | 1.055 | 1.402 | 6.791 | 0.288 | 5.439 | 1.196 |
| 1LM8 | VHL | Protein/Peptide | 0.524 | 16 | 0.375 | 50.421 | 0.704 | 0.644 | 0.91 | 0.419 | 1.188 | 0.353 | 0.461 | 2.233 | 0.297 | 2.325 | 0.918 |
| 1LBQ | VHL | Protein/Peptide | 0.518 | 8 | 0.457 | 25.725 | 0.758 | 0.627 | 0.882 | 1.242 | 0.569 | 2.18 | 0.751 | 7.968 | 0.411 | 5.124 | 0.737 |
| 4AJY | VHL | Protein/Peptide | 0.664 | 37 | 0.615 | 98.441 | 0.73 | 0.61 | 0.802 | 0.475 | 0.99 | 0.48 | 0.276 | 1.304 | 0.32 | 1.532 | 0.387 |
| 6BVB | VHL | Protein/Peptide | 0.671 | 38 | 0.651 | 92.267 | 0.741 | 0.561 | 0.738 | 0.522 | 0.812 | 0.643 | 0.244 | 1.276 | 0.185 | 1.387 | 0.477 |
| 6I7Q | VHL | Protein/Peptide | 0.672 | 29 | 0.66 | 88.151 | 0.726 | 0.593 | 0.791 | 0.764 | 0.621 | 1.23 | 0.596 | 2.273 | 0.226 | 2.431 | 1.383 |
| 1PY2 | IL-2 | Ligand | 0.691 | 30 | 0.709 | 43.218 | 0.639 | 0.556 | 0.839 | 1.832 | 0.428 | 4.282 | 1.807 | 5.104 | 0.221 | 6.326 | 0.838 |
| 1PW6 | IL-2 | Ligand | 0.741 | 31 | 0.743 | 64.827 | 0.563 | 0.644 | 0.978 | 2.24 | 0.511 | 4.385 | 1.972 | 8.321 | 0.265 | 6.769 | 0.646 |
| 1QVN | IL-2 | Ligand | 0.792 | 55 | 0.804 | 108.045 | 0.684 | 0.586 | 0.829 | 1.183 | 0.75 | 1.577 | 1.322 | 7.925 | 0.371 | 5.8 | 1.071 |
| 1M49 | IL-2 | Ligand | 0.717 | 28 | 0.72 | 54.194 | 0.594 | 0.627 | 0.93 | 2.218 | 0.47 | 4.721 | 1.45 | 7.101 | 0.483 | 5.559 | 0.793 |
| 1M48 | IL-2 | Ligand | 0.715 | 35 | 0.714 | 62.426 | 0.624 | 0.596 | 0.825 | 1.714 | 0.614 | 2.793 | 1.04 | 8.687 | 0.2 | 5.954 | 0.911 |
| 4NEJ | IL-2 | Ligand | 0.52 | 15 | 0.444 | 48.706 | 0.789 | 0.601 | 0.906 | 0.731 | 0.86 | 0.85 | 1.529 | 1.634 | 0.327 | 1.356 | 0.746 |
| 3QB1 | IL-2 | Apo | 0.775 | 24 | 0.74 | 47.334 | 0.442 | 0.796 | 1.273 | 2.679 | 0.607 | 4.41 | 1.274 | 9.057 | 0.38 | 7.779 | 1.295 |
| 3INK | IL-2 | Apo | 0.434 | 10 | 0.367 | 25.725 | 0.855 | 0.512 | 0.613 | 0.516 | 0.729 | 0.709 | 1.034 | 3.174 | 0.5 | 4.785 | 1.436 |
| 1M47 | IL-2 | Apo | 0.475 | 19 | 0.353 | 40.817 | 0.736 | 0.532 | 0.745 | 0.305 | 1.154 | 0.264 | 0.265 | 9.218 | 0.325 | 8.434 | 1.271 |
| 1M4C | IL-2 | Apo | 0.64 | 25 | 0.599 | 54.88 | 0.583 | 0.625 | 0.918 | 1.402 | 0.755 | 1.857 | 0.887 | 9.549 | 0.172 | 8.407 | 0.988 |
| 1Z92 | IL-2 | Protein/Peptide | 0.531 | 18 | 0.447 | 40.474 | 0.69 | 0.607 | 0.907 | 0.764 | 0.967 | 0.79 | 1.893 | 4.319 | 0.229 | 4.626 | 0.575 |
| 2B5I | IL-2 | Protein/Peptide | 0.518 | 10 | 0.469 | 24.01 | 0.73 | 0.587 | 0.927 | 2.14 | 0.554 | 3.863 | 1.817 | 9.338 | 0.115 | 8.601 | 1.295 |
| 2ERJ | IL-2 | Protein/Peptide | 0.603 | 26 | 0.597 | 44.247 | 0.748 | 0.506 | 0.599 | 0.609 | 0.569 | 1.069 | 2.635 | 3.803 | 0.294 | 4.174 | 1.692 |
| 5M5E | IL-2 | Protein/Peptide | 0.548 | 21 | 0.495 | 37.73 | 0.731 | 0.552 | 0.786 | 0.453 | 0.82 | 0.552 | 1.213 | 7.565 | 0.376 | 6.656 | 0.757 |
| 4KJU | XIAP | Ligand | 0.638 | 30 | 0.434 | 71.687 | 0.6 | 0.652 | 1.063 | 0.029 | 1.45 | 0.02 | 0.814 | 5.668 | 0.301 | 4.763 | 0.656 |
| 4KJV | XIAP | Ligand | 0.59 | 28 | 0.498 | 69.286 | 0.654 | 0.601 | 0.939 | 0.082 | 1.106 | 0.074 | 1.192 | 4.834 | 0.23 | 4.136 | 0.775 |
| 4KMP | XIAP | Ligand | 0.602 | 29 | 0.528 | 65.513 | 0.663 | 0.609 | 0.885 | 0.32 | 1.055 | 0.303 | 1.12 | 6.809 | 0.387 | 6.455 | 0.776 |
| 4HY0 | XIAP | Ligand | 0.588 | 27 | 0.545 | 61.397 | 0.722 | 0.551 | 0.721 | 0.321 | 0.839 | 0.382 | 1.269 | 5.329 | 0.429 | 5.091 | 0.876 |
| 6H6Q | XIAP | Ligand | 0.61 | 32 | 0.556 | 63.112 | 0.722 | 0.575 | 0.814 | 0.401 | 0.983 | 0.407 | 1.351 | 3.887 | 0.299 | 3.121 | 0.353 |
| 6H6R | XIAP | Ligand | 0.613 | 28 | 0.551 | 70.658 | 0.671 | 0.613 | 0.887 | 0.39 | 0.965 | 0.404 | 1.211 | 4.6 | 0.296 | 3.958 | 0.627 |
| 5C0K | XIAP | Ligand | 0.56 | 21 | 0.46 | 68.6 | 0.708 | 0.635 | 0.942 | 0.344 | 1.077 | 0.319 | 1.204 | 2.051 | 0.246 | 1.791 | 0.708 |
| 5C0L | XIAP | Ligand | 0.609 | 24 | 0.563 | 76.146 | 0.739 | 0.6 | 0.865 | 0.407 | 0.789 | 0.516 | 0.939 | 0.873 | 0.175 | 1.072 | 0.654 |
| 5C3H | XIAP | Ligand | 0.569 | 19 | 0.385 | 67.914 | 0.678 | 0.672 | 1.011 | 0.263 | 1.313 | 0.2 | 1.325 | 3.308 | 0.318 | 2.667 | 1.11 |
| 5C3K | XIAP | Ligand | 0.602 | 29 | 0.521 | 72.03 | 0.642 | 0.608 | 0.884 | 0.326 | 1.074 | 0.303 | 1.094 | 2.299 | 0.44 | 2.073 | 0.777 |
| 5C7B | XIAP | Ligand | 0.566 | 24 | 0.487 | 72.03 | 0.711 | 0.608 | 0.945 | 0.326 | 1.038 | 0.314 | 1.45 | 2.688 | 0.297 | 1.965 | 0.748 |
| 5C7A | XIAP | Ligand | 0.611 | 29 | 0.494 | 66.885 | 0.663 | 0.622 | 0.898 | 0.163 | 1.182 | 0.138 | 1.246 | 3.071 | 0.328 | 2.016 | 0.857 |
| 5C7D | XIAP | Ligand | 0.627 | 32 | 0.539 | 65.17 | 0.652 | 0.616 | 0.899 | 0.318 | 1.112 | 0.286 | 1.345 | 2.854 | 0.448 | 2.212 | 0.629 |
| 5C7C | XIAP | Ligand | 0.826 | 67 | 0.797 | 164.983 | 0.673 | 0.636 | 0.897 | 0.338 | 1.087 | 0.311 | 0.844 | 4.102 | 0.29 | 1.582 | 2.476 |
| 5C83 | XIAP | Ligand | 0.843 | 68 | 0.846 | 156.751 | 0.655 | 0.624 | 0.889 | 0.392 | 0.931 | 0.421 | 1.04 | 3.542 | 0.175 | 1.827 | 2.598 |
| 5C84 | XIAP | Ligand | 0.675 | 35 | 0.639 | 83.692 | 0.676 | 0.611 | 0.82 | 0.424 | 0.872 | 0.486 | 1.415 | 2.757 | 0.283 | 1.918 | 0.388 |
| 5M6F | XIAP | Ligand | 0.914 | 80 | 0.929 | 180.761 | 0.581 | 0.653 | 1.018 | 0.92 | 0.931 | 0.989 | 0.771 | 5.031 | 0.33 | 2.371 | 3.665 |
| 5M6H | XIAP | Ligand | 0.881 | 76 | 0.864 | 181.79 | 0.585 | 0.661 | 1.028 | 0.567 | 1.081 | 0.524 | 0.925 | 4.374 | 0.289 | 2.596 | 3.066 |
| 6EY2 | XIAP | Ligand | 0.57 | 27 | 0.489 | 58.31 | 0.667 | 0.582 | 0.818 | 0.298 | 1.07 | 0.279 | 1.554 | 5.233 | 0.25 | 5.156 | 1.364 |
| 5OQW | XIAP | Ligand | 0.551 | 25 | 0.474 | 62.426 | 0.745 | 0.575 | 0.812 | 0.196 | 1.045 | 0.187 | 1.607 | 4.521 | 0.305 | 4.665 | 0.406 |
| 5M6E | XIAP | Ligand | 0.791 | 52 | 0.803 | 132.398 | 0.706 | 0.599 | 0.813 | 0.927 | 0.718 | 1.292 | 1.901 | 2.008 | 0.074 | 1.846 | 1.023 |
| 5M6L | XIAP | Ligand | 0.667 | 38 | 0.62 | 75.803 | 0.664 | 0.607 | 0.875 | 0.4 | 0.993 | 0.402 | 0.936 | 4.055 | 0.153 | 3.279 | 0.615 |
| 5M6M | XIAP | Ligand | 0.597 | 25 | 0.521 | 77.861 | 0.671 | 0.635 | 0.936 | 0.342 | 1.011 | 0.338 | 1.628 | 4.511 | 0.386 | 3.778 | 0.588 |
| 3HL5 | XIAP | Ligand | 0.561 | 26 | 0.484 | 57.281 | 0.694 | 0.579 | 0.853 | 0.258 | 1.052 | 0.245 | 2.327 | 5.136 | 0.325 | 3.941 | 0.581 |
| 3EYL | XIAP | Ligand | 0.549 | 22 | 0.477 | 55.223 | 0.735 | 0.582 | 0.835 | 0.217 | 0.958 | 0.227 | 1.979 | 5.588 | 0.442 | 3.85 | 1.243 |
| 4EC4 | XIAP | Ligand | 0.553 | 20 | 0.493 | 65.513 | 0.794 | 0.576 | 0.799 | 0.387 | 0.836 | 0.462 | 1.506 | 10.832 | 0.201 | 9.788 | 1.116 |
| 3G76 | XIAP | Ligand | 0.527 | 19 | 0.442 | 56.595 | 0.712 | 0.597 | 0.962 | 0.405 | 1.002 | 0.404 | 1.879 | 8.709 | 0.411 | 8.113 | 0.58 |
| 3CM2 | XIAP | Ligand | 0.621 | 35 | 0.523 | 63.798 | 0.611 | 0.579 | 0.833 | 0.394 | 1.169 | 0.337 | 1.466 | 6.122 | 0.497 | 3.971 | 0.816 |
| 3CLX | XIAP | Ligand | 0.603 | 28 | 0.428 | 62.769 | 0.646 | 0.621 | 0.9 | 0.013 | 1.355 | 0.01 | 1.307 | 6.053 | 0.27 | 3.544 | 0.677 |
| 3CM7 | XIAP | Ligand | 0.602 | 28 | 0.521 | 55.566 | 0.641 | 0.619 | 0.922 | 0.289 | 1.068 | 0.271 | 1.889 | 5.921 | 0.185 | 4.222 | 0.99 |
| 2JK7 | XIAP | Ligand | 0.586 | 27 | 0.532 | 66.542 | 0.763 | 0.569 | 0.806 | 0.383 | 0.913 | 0.42 | 2.002 | 5.245 | 0.21 | 4.46 | 0.803 |
| 2OPY | XIAP | Ligand | 0.583 | 27 | 0.49 | 66.542 | 0.707 | 0.602 | 0.845 | 0.169 | 1.102 | 0.153 | 1.161 | 5.904 | 0.15 | 3.657 | 0.88 |
| 4J3Y | XIAP | Apo | 0.472 | 17 | 0.386 | 60.025 | 0.837 | 0.542 | 0.736 | 0.032 | 1.004 | 0.032 | 2.017 | 4.778 | 0.177 | 3.98 | 0.639 |
| 2POI | XIAP | Apo | 0.504 | 17 | 0.231 | 53.851 | 0.721 | 0.601 | 0.979 | 0.042 | 1.587 | 0.027 | 2.123 | 5.312 | 0.202 | 4.271 | 0.353 |
| 1NW9 | XIAP | Protein/Peptide | 0.588 | 26 | 0.521 | 54.194 | 0.683 | 0.599 | 0.875 | 0.45 | 0.974 | 0.462 | 1.529 | 5.031 | 0.32 | 3.401 | 0.447 |
| 1G73 | XIAP | Protein/Peptide | 0.524 | 22 | 0.45 | 49.735 | 0.714 | 0.555 | 0.838 | 0.24 | 0.992 | 0.242 | 1.344 | 5.484 | 0.119 | 3.987 | 0.885 |
| 2OPZ | XIAP | Protein/Peptide | 0.58 | 28 | 0.531 | 56.938 | 0.723 | 0.547 | 0.773 | 0.334 | 0.905 | 0.369 | 1.127 | 5.599 | 0.211 | 4.084 | 0.553 |
| 2VSL | XIAP | Protein/Peptide | 0.553 | 23 | 0.489 | 54.88 | 0.736 | 0.567 | 0.813 | 0.249 | 0.927 | 0.269 | 1.97 | 4.884 | 0.24 | 4.273 | 0.512 |
| 2I3H | XIAP | Protein/Peptide | 0.607 | 26 | 0.575 | 55.909 | 0.679 | 0.559 | 0.83 | 0.435 | 0.735 | 0.592 | 1.68 | 5.121 | 0.265 | 3.814 | 0.255 |
| 1OXQ | XIAP | Protein/Peptide | 0.551 | 20 | 0.452 | 59.682 | 0.672 | 0.633 | 1.021 | 0.609 | 1.067 | 0.571 | 1.343 | 5.869 | 0.427 | 4.74 | 0.667 |
| 4J48 | XIAP | Protein/Peptide | 0.537 | 23 | 0.423 | 55.909 | 0.72 | 0.576 | 0.827 | 0 | 1.145 | 0 | 1.265 | 5.114 | 0.32 | 4.782 | 0.333 |
| 4WVS | XIAP | Protein/Peptide | 0.514 | 22 | 0.45 | 58.653 | 0.794 | 0.524 | 0.699 | 0.17 | 0.937 | 0.182 | 1.563 | 4.627 | 0.172 | 4.585 | 0.712 |
| 4WVU | XIAP | Protein/Peptide | 0.54 | 28 | 0.481 | 56.595 | 0.763 | 0.512 | 0.683 | 0.087 | 0.993 | 0.088 | 1.731 | 5.19 | 0.28 | 3.944 | 0.226 |
| 1OXN | XIAP | Protein/Peptide | 0.538 | 21 | 0.461 | 55.566 | 0.731 | 0.582 | 0.881 | 0.481 | 0.977 | 0.493 | 1.161 | 5.681 | 0.439 | 4.731 | 0.707 |
| 1OY7 | XIAP | Protein/Peptide | 0.594 | 27 | 0.542 | 62.426 | 0.686 | 0.575 | 0.83 | 0.486 | 0.894 | 0.543 | 2.059 | 5.764 | 0.454 | 4.644 | 0.616 |
| 4J46 | XIAP | Protein/Peptide | 0.596 | 31 | 0.537 | 57.967 | 0.652 | 0.572 | 0.857 | 0.18 | 1.013 | 0.177 | 1.161 | 5.18 | 0.235 | 3.855 | 0.437 |
| 4J47 | XIAP | Protein/Peptide | 0.599 | 34 | 0.535 | 53.851 | 0.682 | 0.556 | 0.832 | 0.116 | 1.063 | 0.109 | 1.262 | 4.961 | 0.248 | 3.83 | 0.576 |
| 4J44 | XIAP | Protein/Peptide | 0.575 | 29 | 0.515 | 56.938 | 0.707 | 0.556 | 0.786 | 0.098 | 0.997 | 0.098 | 1.84 | 4.736 | 0.171 | 3.723 | 0.739 |
| 4J45 | XIAP | Protein/Peptide | 0.599 | 32 | 0.517 | 60.368 | 0.663 | 0.575 | 0.858 | 0.067 | 1.101 | 0.061 | 1.524 | 4.925 | 0.202 | 3.876 | 0.497 |
| 1SIJ | ZipA | Ligand | ND | ND | ND | ND | ND | ND | ND | ND | ND | ND | ND | ND | ND | ND | ND |
| 1S1S | ZipA | Ligand | ND | ND | ND | ND | ND | ND | ND | ND | ND | ND | ND | ND | ND | ND | ND |
| 1Y2F | ZipA | Ligand | ND | ND | ND | ND | ND | ND | ND | ND | ND | ND | ND | ND | ND | ND | ND |
| 1Y2G | ZipA | Ligand | ND | ND | ND | ND | ND | ND | ND | ND | ND | ND | ND | ND | ND | ND | ND |
| 1F46 | ZipA | Apo | ND | ND | ND | ND | ND | ND | ND | ND | ND | ND | ND | ND | ND | ND | ND |
| 1F47 | ZipA | Protein/Peptide | ND | ND | ND | ND | ND | ND | ND | ND | ND | ND | ND | ND | ND | ND | ND |
|  |  |  |  |  |  |  |  |  |  |  |  |  |  |  |  |  |  |
|  |  |  |  |  |  |  |  |  |  |  |  |  |  |  |  |  |  |
|  |  |  |  |  |  |  |  |  |  |  |  |  |  |  |  |  |  |
|  |  |  |  |  |  |  |  |  |  |  |  |  |  |  |  |  |  |
|  |  |  |  |  |  |  |  |  |  |  |  |  |  |  |  |  |  |

**Table S5.** Reference ligand-bound PDB that was superimposed into Apo and Protein/Peptide- bound structures.

| **PPI Complex** | **Protein** | **Reference ligand-bound PDB** |
| --- | --- | --- |
| DCN1/UBC12 | DCN1 | 5V86 |
| Bcl-xL / BAD/BAK | Bcl-xL | 2YXJ |
| HDM2/p53 | HDM2 | 5LAW |
| XDM2/p53 | XDM2 | 4LWT |
| Bcl-2/Bax/BAD | Bcl-2 | 6QGK |
| MDMX/p53 | MDMX | 6Q9Y |
| HPV E2/ HPV E1 | HPV E2 | 1R6N |
| Menin/MLL | Menin | 4GQ3 |
| VHL/HIF-1A | VHL | 3ZRC |
| IL-2/IL-2Rα | IL-2 | 1M48 |
| XIAP/Caspase-9/Smac | XIAP | 6YE2 |
| ZipA/Fitz | ZipA | 1Y2F |

| **Protein** | **Binding Affinity** | | | |
| --- | --- | --- | --- | --- |
|  | **<1 nM** | **1 nM - 1 *μ*M** | **1 *μ*M - 1 mM** | **>1 mM** |
| DCN1 | 0 | 2 | 4 | 0 |
| Bcl-xL | 0 | 6 | 0 | 0 |
| HDM2 | 11 | 40 | 2 | 0 |
| XDM2 | 0 | 6 | 1 | 0 |
| Bcl-2 | 0 | 2 | 0 | 0 |
| MDMX | 0 | 3 | 2 | 0 |
| HPV E2 | 0 | 1 | 0 | 0 |
| Menin | 0 | 24 | 1 | 0 |
| VHL | 0 | 3 | 3 | 0 |
| IL-2 | 0 | 1 | 2 | 0 |
| XIAP | 0 | 18 | 2 | 2 |
| ZipA | 0 | 0 | 0 | 2 |

**Table S6.** Comparison of the binding affinity values across 12 PPI targets.


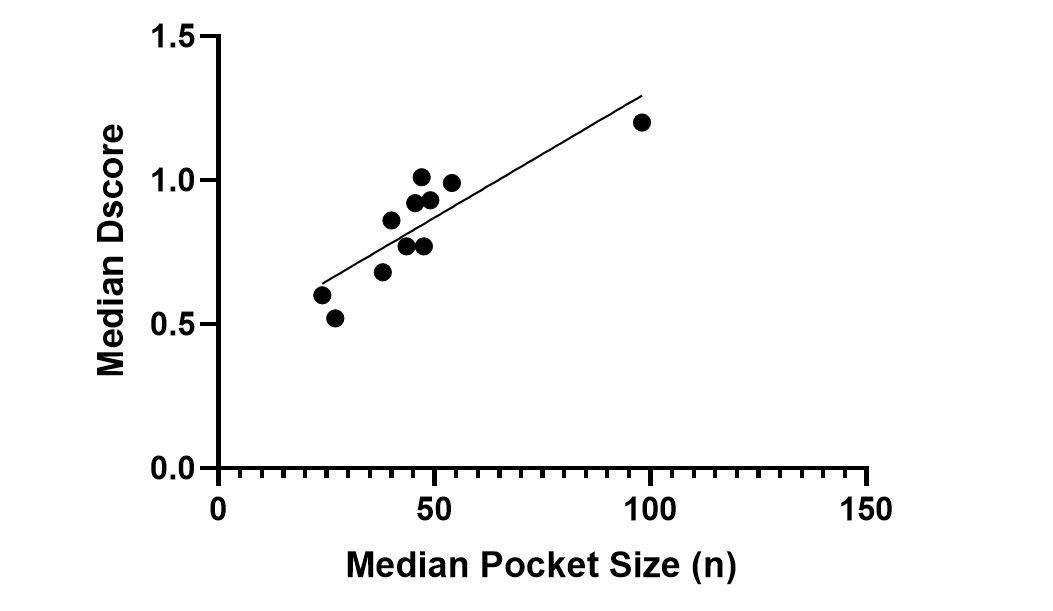
**Figure S1.** Linear regression plots of median Dscore against median pocket size (n), median enclosure factor (e), and median hydrophilicity factor (p).


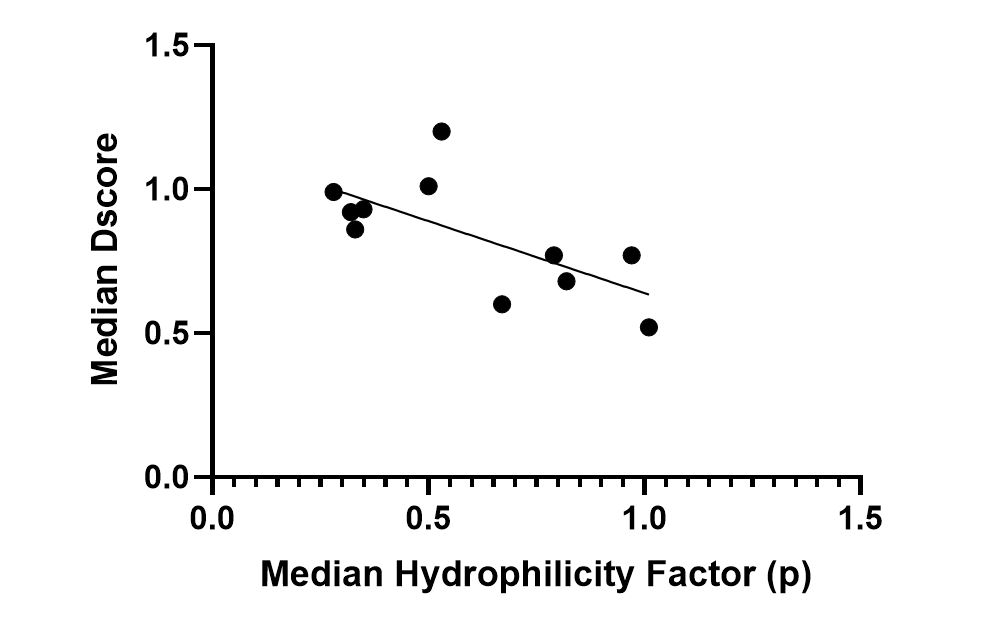

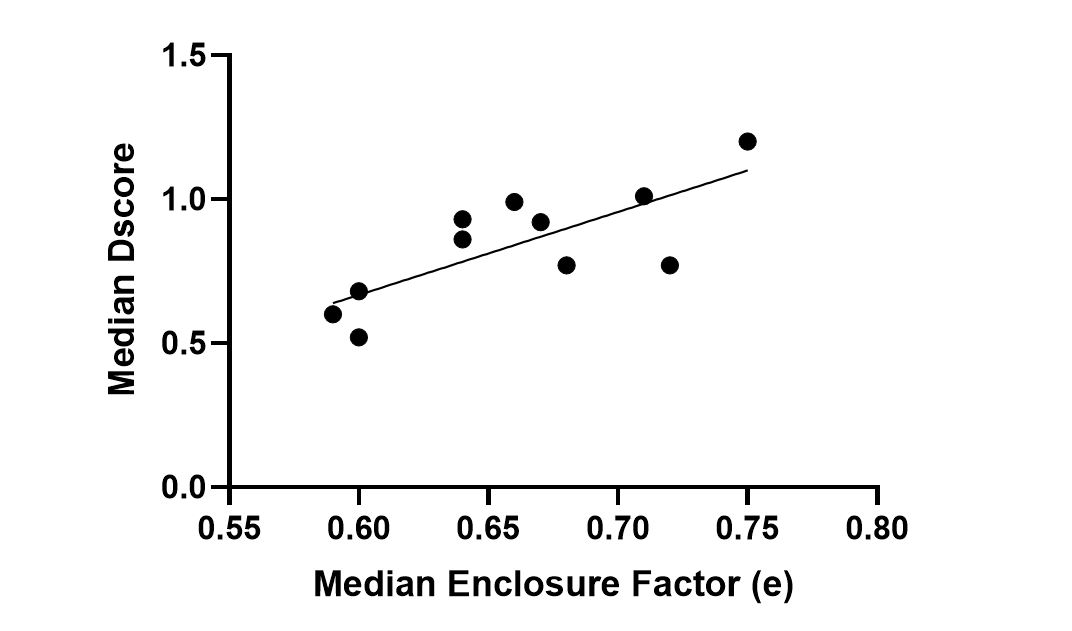

Supplement: Supplementary file 4 — Supplementary Information 4. [file 41598_2022_12105_MOESM4_ESM.docx]
